# Broadly Neutralizing Antibody Responses in a Large Longitudinal Sub-Saharan HIV Primary Infection Cohort

**DOI:** 10.1371/journal.ppat.1005369

**Published:** 2016-01-14

**Authors:** Elise Landais, Xiayu Huang, Colin Havenar-Daughton, Ben Murrell, Matt A. Price, Lalinda Wickramasinghe, Alejandra Ramos, Charoan B. Bian, Melissa Simek, Susan Allen, Etienne Karita, William Kilembe, Shabir Lakhi, Mubiana Inambao, Anatoli Kamali, Eduard J. Sanders, Omu Anzala, Vinodh Edward, Linda-Gail Bekker, Jianming Tang, Jill Gilmour, Sergei L. Kosakovsky-Pond, Pham Phung, Terri Wrin, Shane Crotty, Adam Godzik, Pascal Poignard

**Affiliations:** 1 International AIDS Vaccine Initiative (IAVI) Neutralizing Antibody Center, La Jolla, California, United States of America; 2 Sanford-Burnham Medical Research Institute, La Jolla, California, United States of America; 3 La Jolla Institute for Allergy and Immunology (LIAI), La Jolla, California, United States of America; 4 Department of Medicine, University of California San Diego, San Diego, California, United States of America; 5 IAVI Medical Affairs, New York, New York, United States of America; 6 University of California San Francisco Department of Epidemiology and Biostatistics, San Francisco, California, United States of America; 7 Department of Pathology and Laboratory Medicine, School of Medicine, Emory University, Atlanta, Georgia, United States of America; 8 Rwanda-Zambia HIV Research Group, Lusaka & Ndola, Zambia; 9 Rwanda-Zambia HIV Research Group, Project San Francisco, Kigali, Rwanda; 10 MRC/UVRI Uganda Research Unit on AIDS, Masaka & Entebbe, Uganda; 11 Centre for Geographic Medicine-Coast, Kenya Medical Research Institute, Kilifi, Kenya; 12 Nuffield Department of Medicine, University of Oxford, Oxford, United Kingdom; 13 Kenya AIDS Vaccine Initiative, Nairobi, Kenya; 14 The Aurum Institute, Parktown, Johannesburg, South Africa; 15 Desmond Tutu HIV Center, University of Cape Town, Cape Town, South Africa; 16 University of Alabama Birmingham, Department of Epidemiology and Department of Medicine, Birmingham, Alabama, United States of America; 17 IAVI Human Immunology Laboratory, Imperial College of Science Technology and Medicine, Chelsea & Westminster Hospital, London, United Kingdom; 18 Monogram Biosciences, Laboratory Corporation of America® Holdings, San Francisco California, United States of America; 19 Center for HIV/AIDS Vaccine Immunology and Immunogen Discovery (CHAVI-ID), La Jolla, California, United States of America; 20 Department of Immunology and Microbial Science, The Scripps Research Institute, La Jolla, California, United States of America; University of Zurich, SWITZERLAND

## Abstract

Broadly neutralizing antibodies (bnAbs) are thought to be a critical component of a protective HIV vaccine. However, designing vaccines immunogens able to elicit bnAbs has proven unsuccessful to date. Understanding the correlates and immunological mechanisms leading to the development of bnAb responses during natural HIV infection is thus critical to the design of a protective vaccine. The IAVI Protocol C program investigates a large longitudinal cohort of primary HIV-1 infection in Eastern and South Africa. Development of neutralization was evaluated in 439 donors using a 6 cross-clade pseudo-virus panel predictive of neutralization breadth on larger panels. About 15% of individuals developed bnAb responses, essentially between year 2 and year 4 of infection. Statistical analyses revealed no influence of gender, age or geographical origin on the development of neutralization breadth. However, cross-clade neutralization strongly correlated with high viral load as well as with low CD4 T cell counts, subtype-C infection and HLA-A*03(-) genotype. A correlation with high overall plasma IgG levels and anti-Env IgG binding titers was also found. The latter appeared not associated with higher affinity, suggesting a greater diversity of the anti-Env responses in broad neutralizers. Broadly neutralizing activity targeting glycan-dependent epitopes, largely the N332-glycan epitope region, was detected in nearly half of the broad neutralizers while CD4bs and gp41-MPER bnAb responses were only detected in very few individuals. Together the findings suggest that both viral and host factors are critical for the development of bnAbs and that the HIV Env N332-glycan supersite may be a favorable target for vaccine design.

## Author Summary

Understanding how HIV-1-broadly neutralizing antibodies (bnAbs) develop during natural infection is essential to the design of an efficient HIV vaccine. We studied kinetics and correlates of neutralization breadth in a large sub-Saharan African longitudinal cohort of 439 participants with primary HIV-1 infection. Broadly nAb responses developed in 15% of individuals, on average three years after infection. Broad neutralization was associated with high viral load, low CD4+ T cell counts, virus subtype C infection and HLA*A3(-) genotype. A correlation with high overall plasma IgG levels and anti-Env binding titers was also found. Specificity mapping of the bnAb responses showed that glycan-dependent epitopes, in particular the N332 region, were most commonly targeted, in contrast to other bnAb epitopes, suggesting that the HIV Env N332-glycan epitope region may be a favorable target for vaccine design.

## Introduction

The humoral immune response to HIV-1 infection comprises in a subset of individuals broad and potent neutralizing antibodies (bnAbs) [1–6]. The elicitation of such Abs prior to infection would presumably protect against infection by most circulating HIV strains and is thus considered one of the highest priorities of the HIV vaccine research field [7–10]. However, thus far, no vaccine candidate has been successful at eliciting bnAbs. Therefore, understanding the development of bnAbs and the clinical, immunological and virological correlates of their elicitation during natural infection is likely to be crucial for the design of a protective vaccine [11,12].

Broadly nAb responses usually develop after 2 to 4 years of HIV infection, in 10 to 20% of individuals [13–21]. Development of neutralization breadth has been mainly associated with high viral load and low CD4+T cell counts [17–20,22]. An association with greater viral diversity in the *env* coding region at early time-points after infection has also been reported [13,18,23] and particular viral sequences or features may favor the emergence of bnAb responses [24]. However, the contribution of parameters such as HIV subtype, host genetic background and immune factors is less documented [25], mostly due to the small numbers of participants, lack of adequate longitudinal sampling and of geographic and demographic diversity in most cohorts studied to date. Furthermore, while an increasing number of studies have focused on the detailed mapping of broadly neutralizing specificities and shown that bnAbs mainly target 5 regions of Env: the CD4 binding-site (CD4bs), the V3-high mannose patch, the V2 apex, the gp41 MPER and the gp120/gp41 interface [26,27], it still remains to be determined whether these different specificities follow similar developmental pathways in all individuals.

To better understand the process leading to the development of bnAbs in natural infection, and identify broadly neutralizers for further in depth longitudinal studies, we studied clinical and immunological correlates of breath development and mapped the specificity of the bnAb responses in the IAVI Protocol C cohort, the largest (N = 439) and most diverse longitudinal primary infection cohort studied to date for heterologous neutralization.

## Results

### Development of broadly neutralizing antibody responses over the course of natural infection

The IAVI Protocol C recruited for longitudinal follow up 613 participants with documented acute and very early HIV-1 infection (Material and Methods), through 9 clinical research centers in Kenya, Rwanda, South Africa, Uganda, and Zambia (S1A Fig in S1 Text). Participants were characterized in terms of demographics, HIV infection risk factors, clinical history, CD4 counts, viral load and disease progression, as well as HLA genotype [28,29] (S1B-D Fig in S1 Text, S1 Table in S1 Text). For the present study, 439 eligible participants were evaluated for plasma neutralizing activity starting at month 24 post-estimated date of infection (EDI) (S1E Fig in S1 Text, Material and Methods), using a 6 cross-clade pseudovirus panel predictive of neutralization breadth on larger panels [16], which previously allowed the identification of IAVI Protocol G elite neutralizers and isolation of potent and broad nAbs [1,2,30] (Material and Methods). Neutralization was measured in 2220 unique samples (1–12 samples per donor, mean = 5.0) representing a mean follow-up of 49.4 months post infection (mpi) (range 24–90 months). Among the 439 participants, 228 (52%) were tested at least up to 48 months post-EDI (mean 64.3 months, range 48–90 months) (S2A-B Fig in S1 Text), defining the M48+ subset.

A neutralization score taking into account both breadth and potency was assigned to each plasma sample tested, as previously described [16] (Material and Methods), and used to rank each participant based on the peak of breadth (S3A Fig in S1 Text). As expected, the score on the 6-virus panel was significantly correlated with neutralization breadth on a medium-sized in-house panel (N = 37) and on a larger reference virus panel [31] (N = 105) (S4 Fig in S1 Text). A score ≥1 approximately predicted a breadth ≥50% on the large 105-virus panel.

**Fig 1:**
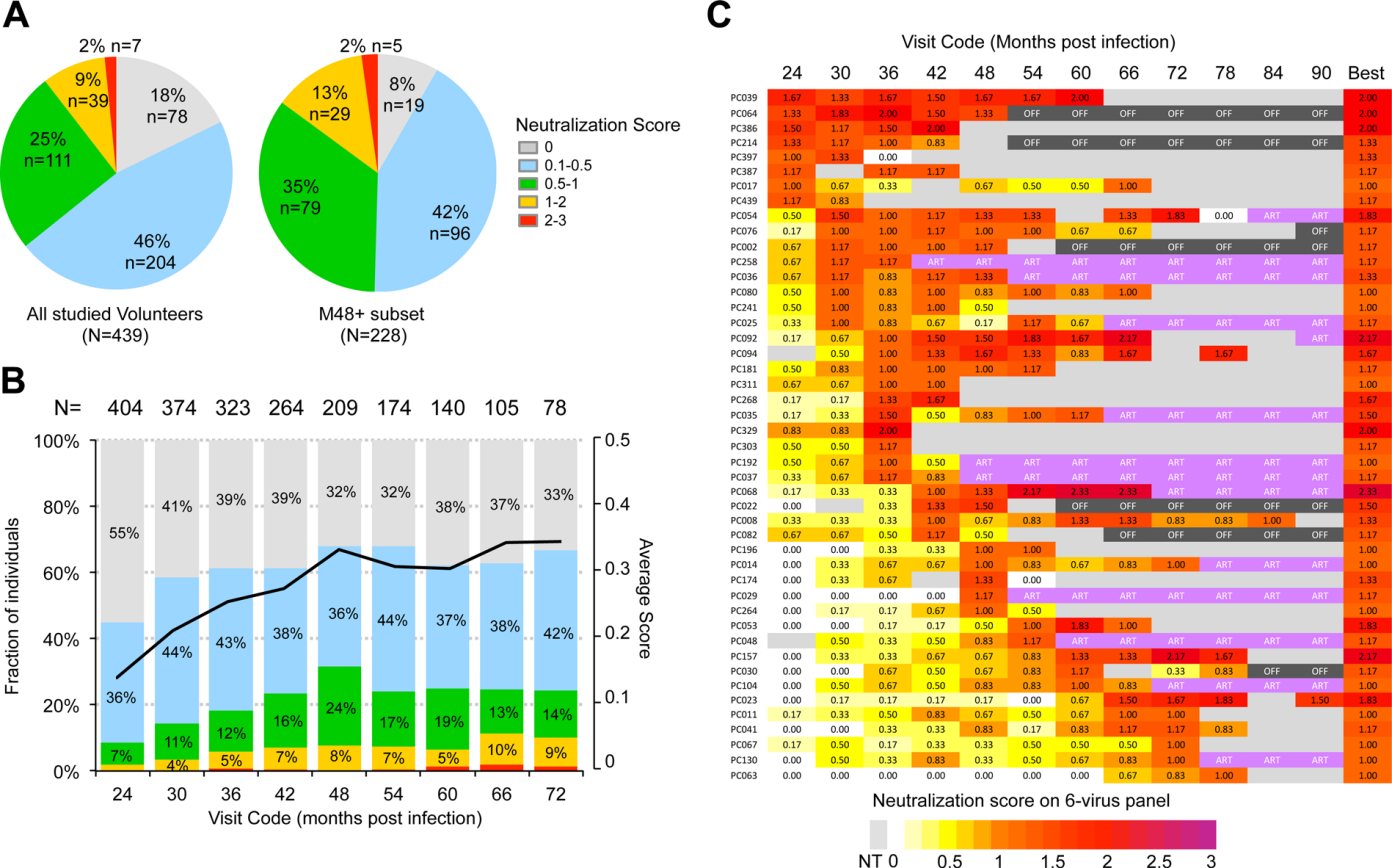
Evolution of broadly neutralizing antibody responses in plasma from Protocol C participants. (A-C) Plasma from HIV-1 infected participants collected at various time points post infection were assessed for neutralizing activity on a predictive 6v-panel [16]. Neutralization score on the 6v-panel was calculated as indicated in Material and Methods (A) Best neutralization score across all time points tested for Protocol C participants. (B) Fraction of Protocol C individuals with the indicated plasma neutralization score at the indicated visits. Neutralization score is color-coded as indicated in (A). (C) Detailed evolution of neutralization score over time (months) for individual Protocol C best neutralizers (N = 46), organized by time to reach neutralization score ≥ 1 in months post infection (MPI). NT: Not Tested. ART: Participants was on Anti-Retroviral Therapy at this visit, OFF: Participant was off-study at this visit.

Overall, 11% (46/439) of study participants achieved broad neutralization (score ≥1) at some point during the course of infection, including 7 individuals with a score ≥ 2. Twenty-five percent (111/439) of participants also acquired moderate neutralization breadth (score ≥0.5 and <1) (Fig 1A). While nearly half of the participants (204/439) displayed low neutralization breath (0 < score < 0.5), a small fraction (8%, 35/439) did not neutralize any virus on the panel. When focusing on the M48+ subset, we found that 15% (36/228) of participants reached a score ≥1, while 35% (79/228) reached a score ≥ 0.5 and <1, as expected from exclusion of participants who might not have reached their best level of neutralization yet.

At month 24 post-EDI, when we started assessing neutralization, only a small subset of individuals had developed breadth, with a score ≥1 in 1–2% participants, confirming that early development of broadly neutralizing responses is rare. In the overall cohort, the average neutralization score further increased gradually over time to peak at month 48 post-EDI, and appeared to plateau or only increase incrementally thereafter (Fig 1B). As the participants did not always comply with visit schedule, we were unable to systematically test all of them for the same time points. Nonetheless, limiting the analysis to participants all tested for the same visits gave virtually identical results (S3B Fig in S1 Text). Similarly, at an individual level, high neutralization scores (≥1) were typically achieved between 2 and 4 years post-infection (mean 3.5 years, range 24–78 months) (Fig 1C, S3A Fig in S1 Text). In most individuals reaching a score of 1 or greater, further neutralizing activity either plateaued or decreased (Fig 1C, S3C Fig in S1 Text). We identified 7 neutralizers in the elite/sub-elite category (scores ≥2) over the study period (Fig 1C).

### Correlates of broadly neutralizing antibody development

We then conducted a thorough statistical analysis to identify potential associations between the development of bnAb responses and a number of clinical parameters (Material and Methods). As suggested by the kinetics of breadth development described above, time from EDI and number of time points tested were significantly associated with best neutralization score (S5A Fig in S1 Text). For the M48+ subset, this association was no longer significant (S5A Fig in S1 Text) and we therefore restricted our further analysis to these individuals, in order to improve accuracy by excluding as much as possible participants for whom follow up time was not long enough to permit breadth development.

**Fig 2:**
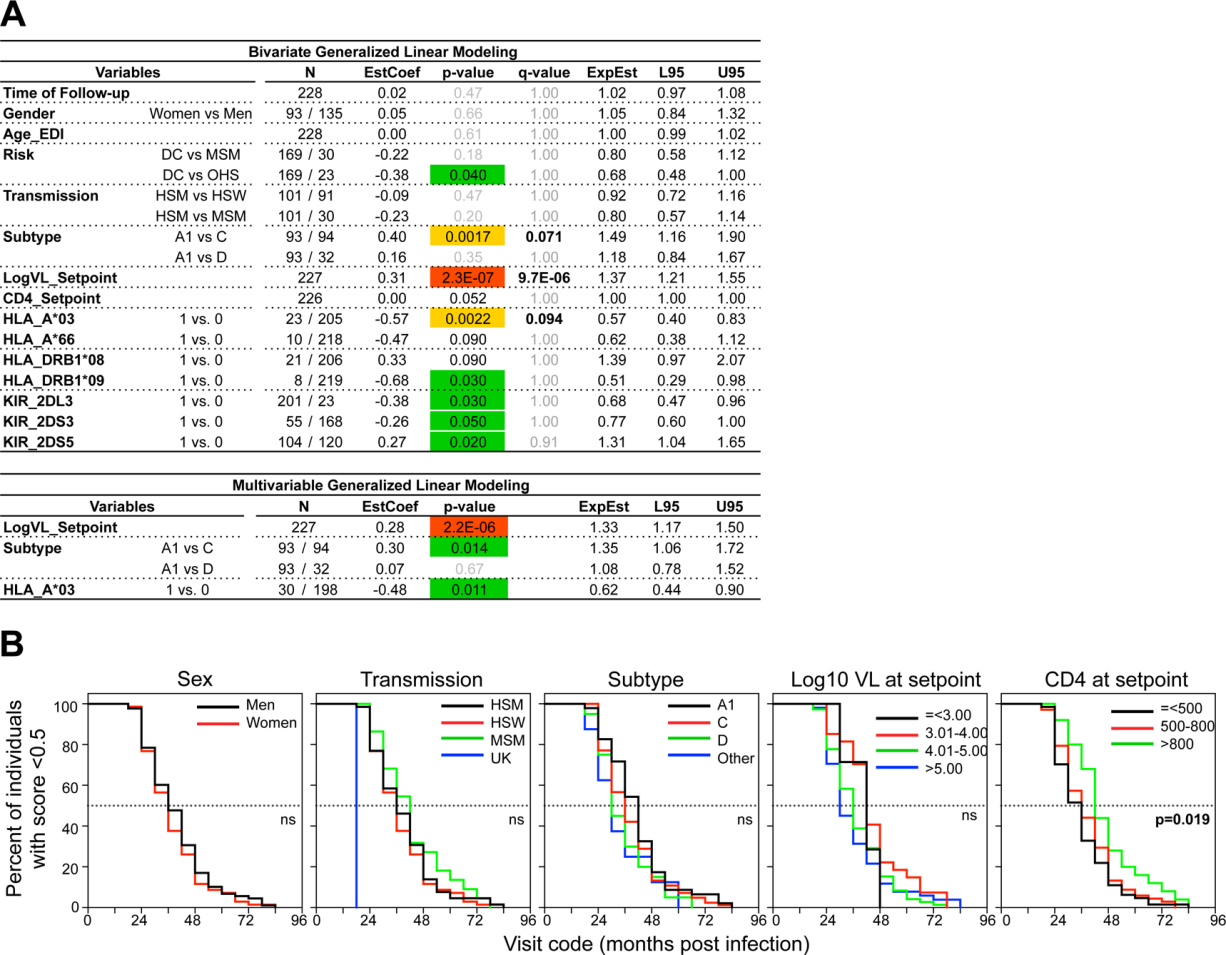
Correlation between clinical parameters and development of broadly neutralizing antibody responses. (A) Bivariate and multivariable GLM correlation analyses between the listed variables, and the best neutralization score for the M48+ subset of Protocol C participants. Number of participants in each subgroup (N) is indicated. Estimated coefficients (EstCoef), p-values, q-values, odd ratios (ExpEst) upper (U95) and lower (L95) values of the 95% confidence interval are indicated. P-values are color coded as follows: 0.01< p-value < 0.05, in green; 0.001< p-value < 0.01, in yellow; 2E-16 < p-value <0.001, in red. Q-values below 0.1 are indicated in bold. (B) Kaplan Meier curves recording the time for Protocol C neutralizers (best neutralization score ≥ 0.5, N = 157) within the indicated subgroups to reach a neutralization score ≥ 0.5. Log-Rank test p-values are indicated. DC: Discordant couple, OHS: Other Heterosexual transmission, HSM: Women to Men Heterosexual transmission, HSW: Men to Women Heterosexual transmission, MSM: Men who have Sex with Men.

A Generalized Linear Model (using a Gamma distribution with Log link function) was chosen to model neutralization scores, which are positive-valued. Country was excluded in favor of recruitment site and subtype as they were found to be highly correlated (country of origin and recruitment center (ρ = 0.85, p≈0); country of origin and infectious subtype (ρ = 0.90, p≈0)). Given the high number of variables, p-values from bivariate analyses were adjusted for False Discovery Rate (FDR) and only parameters with q-values <0.1 were selected for further analyses. The bivariate GLM analyses revealed that set-point viral load, HIV-1 subtype and HLA-A*03 genotype were significantly associated with the neutralization score (Fig 2A, S5B-C Fig in S1 Text). In contrast, age at time of infection, sex, mode of transmission, recruitment site, other HLA and KIR types, and CD4+ T cell count at set-point were not significantly associated (Fig 2A, S5B-C Fig in S1 Text). The neutralization score was further significantly associated with viral load at any visit from month 6 to 48 post-EDI and with the area under curve (AUC) for viral loads between month 6 and 48 (S5D Fig in S1 Text). In contrast to viral load, CD4 T cell counts were inversely associated with neutralization score, and only past 6 months post-EDI, although there was a trend for an association at setpoint and month 6 post-EDI. An inverse association between CD4_AUC and score was also detected. A multivariable GLM analysis further showed that viral load at setpoint remained strongly correlated with neutralization breadth while HIV subtype C and HLA-A*03 genotype became barely significant (Fig 2A).

To evaluate the impact of clinical parameters on the kinetics of bnAb response development, we finally compared the time post-infection necessary to reach various levels of neutralization score across different subgroups, using Kaplan-Meier curves with Log-rank test (Fig 2, S6 Fig in S1 Text). A significant difference was found only for CD4 T cell count at setpoint, individuals with lower CD4 counts developing neutralization score ≥ 0.5 faster than individuals with high CD4 counts (Fig 2). No difference was observed between subgroups for the time to reach broad (score ≥ 1) neutralization. However, the number of individuals included in the latter analysis was very limited.

**Fig 3:**
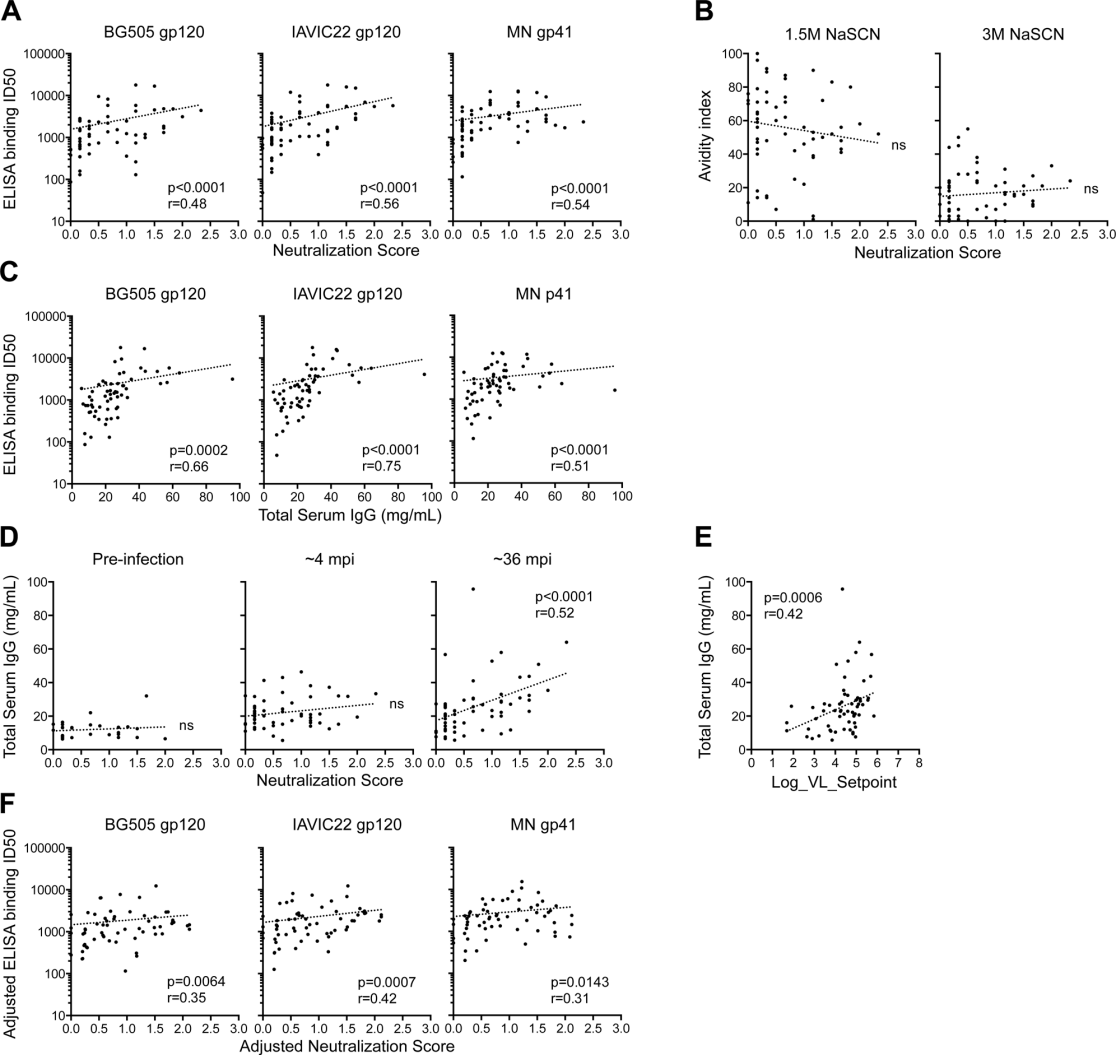
Correlation between total and antigen-specific IgG responses and development of broadly neutralizing antibody responses. Correlations were assessed by Spearman analyses: p-values and r-values are indicated; (ns) not significant. Linear, semi-Log or Log-log regressions are also shown as dotted lines. Neutralization score corresponds to a participant’s best neutralization score on the 6-virus panel across all tested time points. (A,C) IgG binding activity to recombinant MN gp41 (Subtype B), BG505 gp120 (subtype A) and IAVIC22 gp120 (Subtype B) was assessed, by ELISA, in plasma samples of Protocol C participants (N = 61) from the M48+ subset, at visits matching development of bnAb responses (M24-72, mean = 36.6 mpi). (B) Avidity index for IAVIC22-gp120 IgG titers were calculated from high salt (1.5M or 3M NaSCN) ELISA experiments. (C) Total IgG titers were assessed by ELISA. (D) Total IgG titers in pre-infection (N = 27), ~4mpi (N = 56, M00, mean = 4.0 mpi) and ~36mpi (N = 61) M24-72) samples. (E) Total IgG titers in ~36mpi samples (M24-72). (F) ELISA binding ID50 and neutralization score from (A) were standardized to a reference concentration of 20mg/mL of total plasma IgG.

We recently showed, studying the same cohort, that individuals who develop a bnAb response have significantly higher percentages of circulating PD-1+CXCR3−CXCR5+ memory Tfh cells, suggesting that these individuals may be intrinsically more prone to mount antibody responses of greater quality [32]. Therefore, we studied whether this association may be reflected in greater binding titers to HIV Env. As shown in Figs 3A and S7A Fig in S1 Text, neutralization scores were strongly correlated with plasma anti-gp120, -gp41 and–p24 IgG binding ELISA titers in samples from time points matching development of bnAb responses (M24-72, median = 36mpi). No correlation was found between score and anti-gp120 IgG avidity index (Fig 3B, S7A Figure in S1 Text), suggesting that the greater binding titers found in broad neutralizers may not correspond to responses of greater affinity but to a quantitative rather than qualitative difference in the Ab response. We thus looked at potential associations with total plasma Ig titers and found that anti-gp120 and -gp41 titers as well as neutralization score were strongly correlated with total plasma IgG titers in these individuals (Fig 3C and 3D). Correspondingly, the data showed that total plasma IgG titers correlated with VL at set-point (Fig 3E). As previously reported, anti-Gag p24 IgG responses did not correlate with total plasma IgG levels and negatively correlated with viral load (S7B Fig in S1 Text) [33].

As anti-gp120/gp41 ELISA binding titers and neutralization scores were correlated with the total IgG concentration, we normalized both values to the latter (Material and Methods). The neutralization scores were still significantly associated with higher anti-Env IgG binding titers when using adjusted values, and also found to be negatively correlated with normalized anti-Gag IgG responses (Fig 3F, S7C Fig in S1 Text). Greater gp120/gp41 binding titers may be explained by a higher concentration of specific anti-gp120/gp41 Abs or by the presence of Abs of greater affinity in broad neutralizers. The avidity index of the anti-gp120/41 responses still did not correlate with the normalized score (S7D Fig in S1 Text), suggesting that the affinity of anti-gp120/gp41 binding Abs is overall not different between strong and weak neutralizers, though this may be confounded by differences in antibody specificities to neutralizing versus non-neutralizing epitopes on gp120 and gp41.

### Mapping broadly neutralizing plasma activities

**Fig 4:**
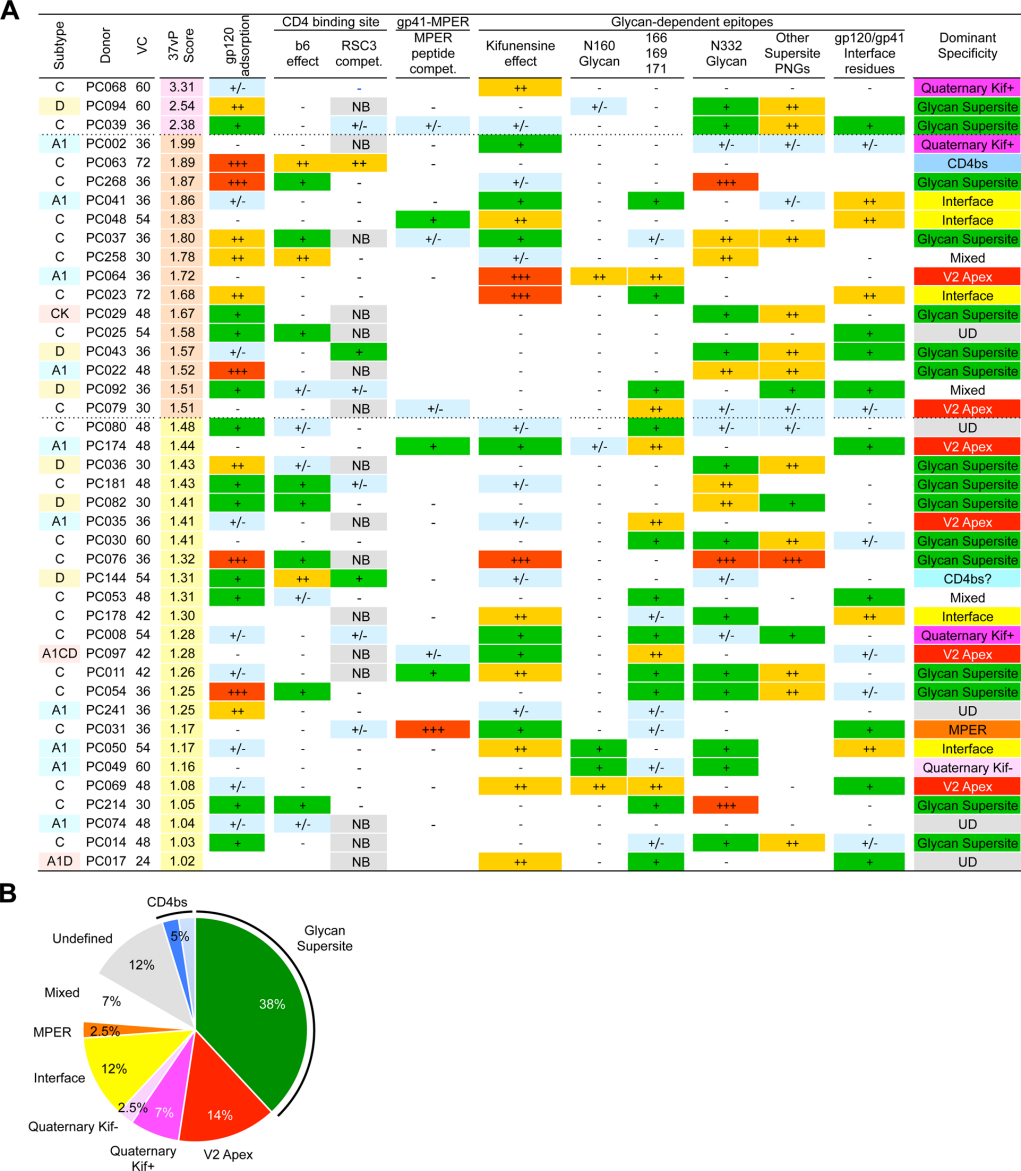
Specificities mediating neutralization breadth and potency in the top 42 Protocol C neutralizers. (A) Samples are ranked by their neutralization score on the 37-virus panel (37vP) (S4 Fig in S1 Text and S2 Table in S1 Text). VC: Visit Code (months post infection). Symbols recapitulate the strength of the phenotypes tested using the different approaches detailed in this manuscript (S7-S11 Figures in S1 Text) to determine the Env epitope region targeted by the plasma broadly neutralizing activity: gp120 absorption of bnAb activity, effect of b6 competition in gp120 absorption experiments, RSC3 binding and neutralization competition, HIV-2 chimera neutralization, viruses produced in presence of kifunensine or bearing mutations. Absent (-), very weak (+/-), weak (+), moderate (++), strong (+++), phenotype was attributed based on i) the median fold or average percent decrease in ID50 and ii) the fraction of viruses which neutralization ID50 was decreased <2 fold, <10 <50 fold or <20%, <40%, <60%, <80%. Blank = not tested, NB: not binding. A dominant specificity was attributed for each sample based on results from all these experiments. UD: Undefined. (B) Overall distribution of dominant nAb specificities mediating plasma neutralization breadth in the top 42 Protocol C neutralizers as detailed in (A).

We then aimed to investigate the antibody specificities associated with broad neutralization in plasma with a score ≥1 on the 37v-panel, corresponding to ≥ 50% breadth on the 105v-panel (n = 42) (S4 Fig in S1 Text, S2 Table in S1 Text). We first asked whether the broadly neutralizing activity of the plasma could be adsorbed on recombinant monomeric gp120 (rgp120). Plasma samples were pre-incubated with rgp120 coated beads or control beads before being tested for neutralization. After verifying by ELISA that all rgp120-binding Abs had been removed, we tested the adsorbed fractions against a cross-clade virus panel (Fig 4A, S8A Fig in S1 Text). About a third of the plasma samples (11/40, 27.5%) were efficiently depleted of broadly neutralizing activity across viruses by adsorption on rgp120, showing that the neutralization breadth was, in those cases, clearly associated with Abs reactive with monomeric gp120. Eleven other samples (27.5%) were only partially depleted of neutralizing activity on rgp120, suggesting the presence of multiple neutralizing Ab specificities in these donors or a partial match with the rgp120 used for the depletion. Finally, samples from 18/40 (45%) participants retained almost complete neutralizing activity after depletion by gp120 monomers, including when rgp120s from different viral strains were used for adsorption, suggesting the presence of quaternary gp120-specific bnAbs or of gp41-specific bnAbs in these plasma.

To investigate the presence of gp41-MPER specific bnAbs, we tested the selected plasma against HIV-2 chimeric pseudoviruses bearing either the full or partial HIV-1 MPER [13,14]. We identified 17 (40%) plasma samples with neutralizing activity against the HIV-2 C1 but not HIV-2 WT, most of them (12/17, 70%) targeting the N-terminus segment (HIV-2 C4) (Fig 4A, S9A Fig in S1 Text). The MPER specific reactivity was further confirmed by competition of the neutralizing activity with an MPER-peptide. Eleven of the 17 plasma samples were competed by this peptide for neutralization of HIV-2 C1 (S9A Fig in S1 Text) but only four (9.5%) plasma (PC048, PC174, PC011, PC031) were further competed for neutralization of several cross-clade HIV-1 pseudoviruses (Fig 4A, S9B Fig in S1 Text) suggesting the presence of bnAbs targeting the MPER. Accordingly, neutralizing activity was not absorbed by rgp120 in these samples (S8A Fig in S1 Text). Peptide competition was particularly strong for participant PC031 suggesting that most of the bnAb activity was directed against the MPER for this individual.

We then assessed in which plasma the presence of CD4 binding site (CD4bs) specific bnAbs may explain the broadly neutralizing activity. We first tested plasma binding activity to the Resurfaced Stabilized Core 3 (RSC3), a probe selective for VRC01-like CD4bs bnAbs, and a mutant (KO-RSC3) with decreased CD4bs bnAbs binding capacity [3,34]. Forty-five percent (19/42) of the plasma specimens tested had differential RCS3/KO-RSC3 reactivity suggesting the possible presence of VRC01-like Abs (S10A Fig in S1 Text). However, RSC3 failed to efficiently compete all but one donor plasma (PC063) for neutralization of a small cross-clade panel of HIV-1 pseudoviruses (Fig 4A, S10B Fig in S1 Text). The results agree with a recent report by Lynch and colleagues showing that although RSC3 binding activity could be found in 47% of HIV-1 infected individuals, RSC3-reactive Abs mediating broad neutralization were only detected in a few individuals [34]. Of note, a competition assay using a different gp120-core molecule, TriMut, that binds, in addition to VRC01-like bnAbs, non-broadly neutralizing CD4bs Abs like F105 or b6 [35] could compete 71% (30/42) of the plasma for neutralization of the CD4bs sensitive strain HxB2 (Fig 4A, S10C Fig in S1 Text) confirming that most HIV-infected individuals develop CD4bs Abs that are not broadly neutralizing. A caveat to the RSC3 competition approach above is that some CD4bs bnAbs may not bind this probe. Therefore, we also performed plasma rgp120 adsorptions in the presence of the non-broadly neutralizing Ab b6 at saturating concentrations [2]. With the exception of three donors, the adsorption on rgp120 of the broad plasma neutralizing activity was not significantly inhibited by the presence of b6 (S8A Fig in S1 Text). In the case of donors PC063 and PC053, b6 greatly competed (>75%) the broad plasma neutralizing activity adsorption for 6/6 and 2/6 viruses, respectively (Fig 4A, S10D and S8A Figs in S1 Text). Interestingly, we also found that neutralization by PC053 and PC063 plasma was enhanced for 5/6 and 3/5 N276A-mutant pseudoviruses, respectively (S4 Table in S1 Text), raising the possibility of the presence of early precursors of VRC01-like CD4bs bnAbs [36]. Together the results confirm that in broad neutralizers, although non-broad CD4bs Abs are common, CD4bs bnAbs are rare.

Broadly nAbs of the PG9 class, that recognize a quaternary epitope in the V2 spike apex region, require the presence of an N-linked glycosylation site at residue 160 and viruses treated with the glycosidase inhibitor kifunensine resist neutralization by PG9-like Abs to a large extent [1,30,37,38]. We found 2 participants (PC064, PC069) with N160K-sensitive plasma neutralizing activity (Fig 4A, S10A Fig in S1 Text). Both PC064 and PC069 plasma also showed a markedly reduced neutralizing activity against kifunensine-treated pseudoviruses and other V2 mutant (residues 166, 169, 171) pseudoviruses (Fig 4A, S11 Fig in S1 Text). Additionally, the broadly neutralizing activity of the PC064 plasma was retained following removal of gp120-specific antibodies through adsorption on rgp120s from 4 different strains (S8A Fig in S1 Text). Together these results strongly suggested the presence of PG9-like bnAbs in donor PC64. In contrast, a small but significant decrease in neutralization by the PC069 plasma was observed after rgp120 absorption, suggesting that in this case the N160 glycan-dependent apex epitope recognized by the bnAbs may be less dependent on quaternary structures than PG9 and displayed on the corresponding rgp120s. Four other samples which neutralizing activity was not depleted by rgp120 but did not depend on the N160 glycan (PC079, PC174, PC035, PC097), were also sensitive to mutations at position 166, 169 and 171, suggesting the presence of bnAbs targeting the apex more similar to the recently described CAP256-VRC26 bnAbs [39].

We found 11 other plasma samples (26%) which neutralizing activity was not depleted by rgp120 not mapping to the apex and affected to various degrees when viruses were treated with kifunensine (Fig 4A, S11A Fig in S1 Text). Four of these samples (PC041, PC048, PC178, PC050) were significantly affected by mutations shown to impact the recently described bnAbs targeting the quaternary gp120/gp41 interface epitope [6,40,41] (Fig 4A, S11B Fig in S1 Text). Interestingly, broad neutralization of PC023 plasma was also affected by these mutations although the broadly neutralizing activity could efficiently be depleted by rgp120. Of the five remaining samples with undefined quaternary-specific bnAb responses, one sample corresponded to the participant with the greatest breadth (PC068) (Fig 4A, S8B Fig in S1 Text), neutralizing 97% of the viruses tested (S4A-B Fig in S1 Text).

A third class of glycan-dependent bnAbs recognizes the high mannose patch centered around the N332 glycan in the V3 region [2,20,30,42,43]. We identified 17 broad neutralizers (40%) whose plasma neutralizing activity was significantly affected by single or double N332 supersite mutations in the context of more than 3 viruses (Fig 4A, S11A fig in S1 Text), often across different subtypes. Interestingly, in half of cases, the N332-glycan dependent bnAb activity could only be slightly depleted by rgp120 adsorption (Fig 4A). Plasma from participants PC076, PC037 and PC011 displayed both sensitivity to the N332A mutation and to kifunensine treatment [44] of various viruses across different clades. Altogether, glycan-dependent neutralization (ie affected by removal of PNG and kifunensine-sensitive) was detected in 60% (25/42) of top Protocol C neutralizers (Fig 4A and 4B).

## Discussion

In the present study, we investigated the development of bnAb responses against HIV-1 in the largest (n = 439) and most diverse longitudinal primary infection cohort studied to date for neutralization. Overall, about 15% of Protocol C participants who were followed for at least 48 months reached a neutralization score ≥1, roughly corresponding to more than 50% breadth on a large 105-virus panel (S4D Fig in S1 Text) a prevalence equivalent to that observed in previously studied cohorts (S12 Fig in S1 Text, Amsterdam, total HIV+ cohort size N = 82; Massachusetts General Hospital, N = 17; CAPRISA, N = 40) [16,19,20,22]. In addition, a moderate neutralization breadth (score = 0.5–1), corresponding to about 20 to 50% breadth on a large virus panel, developed in another third of the donors. This observation is consistent with studies suggesting that some degree of breadth develops in a large proportion of HIV-infected individuals [21,45]. Protocol C participants who had not developed breadth by year 4 were unlikely to do so thereafter as the cohort average neutralization score plateaued at 48 months, with only a slight increase in the proportion of the highest scores afterwards, essentially due to an augmentation in neutralization potency in a few donors. Of all the subjects who developed scores ≥1, only three did so past month 48. While the development of breadth usually takes at least 2 years possibly due to the stochastic nature of the bnAbs maturation process and Env evolution, this general lack of development of broadly neutralizing Ab responses later in infection may reflect an increased disruption of CD4 and B cell responses as the infection progresses, as seen with decreased responses to vaccination [46,47]. While neutralization breadth in the Amsterdam and MGH cohorts, both predominantly composed of subtype B-infected men having sex with men (MSM) participants, was found to emerge between 1–2 years of infection [18,19,22,48], participants in our study developed breadth on average 3 years post-infection, similar to the CAPRISA cohort which is composed of subtype C-infected high-risk women [20]. Differing parameters such as mode of transmission, HIV-1 subtype, host genetics, general health or other concomitant infections could account for the difference between the Caucasian and African cohorts.

In agreement with previous publications, we found the development of neutralization breadth to be most strongly correlated with viral load both at setpoint and all further time points tested [15,17,18,20,23,45]. Viral load is known to be influenced by the host HLA genotype [49–55] and the nature of the transmitted virus [56–59]. Our statistical analysis identified infection by subtype C viruses and HLA-A*03 genotype to be associated with neutralization breadth. However, the relatively low significance of these associations in our multivariate analysis suggests that the correlation may be essentially due to the impact of these parameters on viral load itself [28,60]. Nevertheless, particular phenotypic and genotypic features of subtype C transmitted/founder viruses have been described that could potentially favor the development of neutralization breadth [61–64]. In particular, not all individuals with high viral load developed bnAb responses, suggesting that other factors are at play or, that an earlier disruption of immune responses in some individuals may prevent bnAb development as discussed below.

An association between the development of breadth and low CD4 T cell levels at various time points has been described in some studies [18–20,23,31]. Interestingly, in our cohort, in contrast to the association with high viral load, the development of bnAb responses significantly correlated with low CD4 T cell counts only at later time points, past 6 months of infection (S5D Fig in S1 Text). An intrinsic difference in CD4 levels between donors developing broad neutralization or not cannot be totally ruled out as CD4 levels prior to infection were not available [18], but it is tempting to hypothesize that the VL drives the association with breadth and that the low CD4 levels are merely the reflection of the high viral replication and disease progression [18,22,65].

However although CD4 T cell levels were for the most part lower in individuals developing bnAbs, we have shown recently that Protocol C broad neutralizers have significantly higher relative frequencies of a population of blood memory CD4 Tfh cells [32]. This is consistent with observations from other studies [19,66] and together with the high level of somatic hyper-mutations (SHM) found in most bnAbs, suggests that an intrinsic greater ability to provide help to B cells in certain individuals may favor the generation of highly affinity-matured antibody responses and thus the elicitation of bnAbs. Here we showed that bnAb responses were associated with greater titers of Env (gp120 and gp41) and lower Gag binding Abs in ELISA, even after normalization for total Ig concentration, which as previously shown, also correlated with broad neutralization [67]. The absence of difference in avidity index of anti-Env responses between strong and weak neutralizers suggests that the higher binding titers found in individual with breadth may not be due to the presence of more highly affinity-matured Abs but rather, to a larger diversity of Abs, which may favor the emergence of bnAb responses by exerting cooperative pressure on the virus [68] or by limiting the escape landscape that the virus can explore in response to neutralization. Indeed, a number of escape mutations from nAbs lead to the exposure of epitopes that are usually occluded on the Env trimer and that are the target of Abs elicited by monomeric gp120 and gp160. In this sense, a greater diversity of such Abs may put a pressure on the virus, limiting pathways of escape in a manner that may favor the selection of nAbs targeting conserved regions exposed on the trimer. Further mapping of anti-Env specificities present in weak and broad neutralizers as well as comparison between SMH levels in anti-Env Abs in these donors will help answer these questions.

Taken together, these data suggest a model where a higher level of chronic antigenic stimulation over a prolonged time may lead to the activation of a greater number of naïve B cells, and increase the probability, in a stochastic model, to activate cells bearing a germline BCR more amenable to development into a bnAb lineage. In addition, a high level of antigenic stimulation may further impact the B cell selection process in germinal centers and increase the likelihood of the selection of cells on the path to broad neutralization. Alternatively or concomitantly, a high level of viral replication may lead to a greater or differing stimulation of innate pathways that could favorably impact the humoral response (different “adjuvant effect”, better antigenic presentation). A higher viral load may also contribute to the generation of a greater antigenic diversity (currently under investigation in Protocol C) which may favor the selection of Abs with broadly neutralizing activity, as suggested by some studies [23,39,69,70]. Alternatively, diversity may be the consequence of the elicitation of a broad Ab response [48,71,72]. Both processes could be co-dependent, Env escape mutants being selected in response to neutralizing Abs and a greater diversity of escape mutants leading to the selection of a greater variety of Abs in a cycle increasing the probability of eliciting bnAbs.

In accordance with previous mapping studies, we found that for most Protocol C broad neutralizers, the bnAb activity essentially mapped to one or a limited number of Ab specificities in each individual [2,19,20,73,74]. However, we were unable to clearly map the neutralizing antibody response in about 12% of broad neutralizers suggesting that Abs of not yet known specificity were responsible for the breadth in these donors or, that the bnAb activity in these plasma was due to the presence of Abs of several different specificities [13,68,69,75,76]. The prevalence of each bnAb specificity in our cohort was also in line with other studies [2,13,18–20,22,34,48,74,77–79]. Although most Protocol C top neutralizers developed antibodies to the CD4bs, very few developed broad CD4bs Abs. CD4bs bnAbs usually bear an exceptionally high level of SHM and may require more time to develop than bnAbs targeting other epitopes. In addition, structural constraints and the need to use mainly a unique VH family likely further limit the probability of developing such bnAbs. Accordingly, the Protocol C participant who developed broad neutralization targeting the CD4bs (PC063) appeared to do so relatively late, at month 66 post-EDI [2–4,19,34,74,76,80]. Similar to the CD4bs, only one study participant with a broadly neutralizing response mapped clearly to the gp41-MPER. The proximity to the membrane, the important structural constraints that an antibody needs to circumvent to reach the gp41-MPER and potential self-reactivity issues are likely responsible for the paucity of this type of bnAb response [81].

In contrast, we found that glycan-dependent bnAb specificities (i.e. N332-glycan supersite, V2 Apex, gp120/41 interface, other kifunensin-sensitive specificities) largely dominated (60%) the bnAb responses in top neutralizers in our cohort. Within the glycan specificities, we identified 6 donors (15%) with bnAbs targeting the trimer apex like PG9 (N160-glycan dependency and kifunensine sensitivity) or CAP256-VRC26 bnAbs (sensitivity to 166/169/171 mutations, low dependency on N160-glycan, low sensitivity to kifunensine). The frequency of this type of responses compared to CD4bs or MPER bnAbs responses suggests that the apex may be an interesting vaccine target, particularly in light of the newly developed soluble native Env trimers properly presenting the apex epitopes [82,83]. We also identified several individuals (25%) with bnAb responses that were not adsorbed on rgp120 monomers, with varying levels of kifunensine sensitivity and not mapping to either the MPER nor the trimer apex, similar to the recently described bnAbs PGT151-8, 8ANC195 and 35O22 targeting discrete epitopes the gp120/gp41 interface [6,40,41]. However, mutations previously shown to impact binding of these mAbs, did not result in a clear phenotype suggesting that the bnAbs in these donors, including the best Protocol C neutralizers, may target either yet other discrete epitopes at the gp120/gp41 interface or at the apex, or a novel epitope of the Env trimer. On its own, the N332-glycan region accounted for nearly 40% of the broadly neutralizing specificities in Protocol C. Additionally, some participants had a mixed signal for bnAbs targeting this region (PC002, PC025 PC008, PC080, PC049) suggesting the presence of glycan-specific nAbs of narrower breadth and that N332 supersite Abs are significantly more easily elicited than any other broad specificity. A greater accessibility on the Env spike may explain the higher prevalence for this region, with the ability for Abs to reach from various angles and potentially allowing the use of a number of different Ab gene families. Furthermore Abs to the glycan patch have been shown to be promiscuous in their binding to different glycans of this region which may also favor recognition by a greater number of Abs (although whether this is the cause or the consequence of the elicitation of such antibodies is arguable) [84,85].

Together our findings confirm in a large African cohort with a diverse range of infecting HIV-1 subtypes that a combination of viral and host factors is likely to be necessary for the development of a broadly neutralizing antibody response to HIV-1, explaining why only a fraction of infected individuals develop high levels of such responses. Although in a few individuals neutralization breadth increases over a relatively short time, less than 12 months, in most cases bnAb responses develop around 3 years post-infection, possibly due to the necessity of prolonged antigenic stimulation, and it remains to be seen whether more favorable kinetics may be elicited through efficient vaccination regimens. The development of neutralization breadth may represent a fine balance between a high viral replication needed for antigenic stimulation but leading to a faster decline of the immune system, and the necessity of having immune responses conserved long enough to efficiently elicit bnAbs. As an optimistic note, in healthy individuals the elicitation of bnAbs may not be limited by the crippled immune system found in HIV-infected individuals and may be successful in a larger fraction of individuals. A detailed analysis of the development of bnAb lineages in top neutralizers will help understand which specificities are most amenable to elicitation through vaccination and whether Env evolution pathways associated with specific lineages suggest particular immunogen designs or vaccine strategies. Studying antibody developmental pathways in various individuals sharing the same broad specificity will also be critical, as finding similarities between donors in Env evolution or in the nature of the Env triggering the broad lineage would strongly suggest a path for immunogen design. Our study suggests that the glycan patch surrounding the N332 glycan is the most favorable target for vaccines and should be a high priority for immunogen design.

## Materials and Methods

### Ethics statement

The IAVI-sponsored Protocol C cohort participants were selected through rapid screening of adults with a recent history of HIV exposure for HIV antibodies in Uganda, Rwanda, Zambia, Kenya and South Africa [28]. After obtaining written informed consent, blood samples were collected from HIV-1 infected participants quarterly for the first two years and every 6 months thereafter. The study was reviewed and approved by the Republic of Rwanda National Ethics Committee, Emory University Institutional Review Board, University of Zambia Research Ethics Committee, Charing Cross Research Ethics Committee, UVRI Science and Ethics Committee, Kenyatta National Hospital Ethics and Research Committee, KEMRI Scientific and Ethics Review Unit, University of Cape Town Research Ethics Committee, University of Kwazulu-Natal Biomedical Research Ethics Committee, Mahidol University Ethics Committee, Sanford-Burnham Medical Research Institutional Review Board, Veterans Affairs San Diego Institutional Review Board, LIAI Human Subjects Committee, and Scripps Institutional Review Board.

### Cohort characteristics

Between February 2006 and December 2011, 613 participants were enrolled in Protocol C and over 7,600 time points were sampled for plasma, plasma and PBMCs. Median time from EDI to enrolment was 54 days (mean 81.7 days, range 10 to 396 days). Protocol C participants eligible for this study were age 18 or older, with >24 months of follow-up and antiretroviral therapy (ART) naïve (N = 439; 232 (52%) are still on-study). Overall the participants included in this study (439/613, 72%) were highly representative of the entire cohort population regarding gender, age, mode of transmission, infectious subtype, clinical site, viral load and CD4 T cell count (S1B-D Fig in S1 Text, S1 Table in S1 Text). Visits were scheduled and coded based on the number of months post infection (MPI), calculated from the estimated date of infection (EDI). Visits that deviated from schedule kept the original coding. However, we verified that despite these deviations from the scheduled visit calendar, the mean of adjusted MPIs within each group was in good accordance with the VC and that each VC group was statistically distinct from the previous and the next (S2D Fig in S1 Text). Data were collected at every study visit, including HIV risk behavior (baseline only), demographics, symptom-directed examinations including data on comorbidities and opportunistic infections, CD4 T cell count and viral load. Although enrollment closed in 2011, the longitudinal follow-up continues.

### High throughput neutralization screening

Neutralizing activity in longitudinal Protocol C samples was assessed with a recombinant virus assay (Monogram Biosciences, LabCorp) using a reference panel of full-length *env* of viruses previously selected to stratify infected individuals by the breadth and potency of their nAb response [16]: 92TH021 (CRF0-AE), 94UG103 (Clade A), 92BR020 (Clade B), JRCSF (Clade B), IAVIC22 (Clade C, also named MGRM-C026) and 93IN905 (Clade C). Briefly, pseudoviruses capable of a single round of infection were produced by co-transfection of HEK293cells with a sub-genomic plasmid, pHIV-1lucΔu3, that incorporates a firefly luciferase indicator gene and a second plasmid, PC0XAS that expressed HIV-1 *env* clones. Following transfection, pseudoviruses were harvested and used to infect U87 cell lines expressing CD4 plus the CCR5 and CXCR4 co-receptors. Serial 3-fold dilutions of plasma, starting 1:100, were assessed for neutralization against each of the 6 viruses listed above and NL43, a Tier-1A subtype-B virus, as a positive control. Virus infectivity was determined 72h after inoculation by measuring amount of luciferase activity. Positive neutralization was defined as 50% inhibition of infection of an HIV strain at a 1:100 plasma dilution and when the percent inhibition was at least 1.7 fold higher than percent inhibition against the specificity control, aMLV. The level of neutralizing activity of an individual sample was determined by a neutralization score defined as a weighted average of log-transformed 50% neutralization end point dilutions across the reference pseudoviruses neutralization screening panel and excluding the negative and positive controls, aMLV and NL43 respectively: (*Score = Average (log3 (dilution/100)+1))*. All titers below the limit of detection were assigned a value of 33 for purposes of calculating a neutralization score. Log-transformed values ranged from 0.0 to 4.0 with 0.0 representing a sample with undetectable titers to a given pseudovirus as described previously [16].Samples were equally tested across clinical sites (S1B and S2C Figs in S1 Text).

### Statistics

Statistical analyses were performed using free software R Bioconductor, version 3.0.1 and GraphPad Prism 6. The analysis was performed on the best neutralization score (ranging from 0–2.33) across all time points tested for all Protocol C participants included in this study (N = 439) and the M48+ subset (N = 228). Factors analyzed included participant age at EDI, gender, risk group, viral load (VL, log10-transformed) and CD4 T cell count at setpoint (defined as the first measurement between days 70 and 350 from the estimated of infection), HLA genotype, KIR genotype, infecting HIV subtype, time post-infection of the last time point tested for neutralization (Follow-Up time). A Bivariate Generalized Linear Model GLM) using a Gamma distribution and a Log link function was used to investigate associations between the best neutralization score and each individual clinical parameter. A value of 0.0001 was added to the best neutralization score to avoid zero values. Spearman’s rank correlation, Mann-Whitney U- or t-test, Fisher’s Exact test, and Kruskal-Wallis test or ANOVA were used to examine the associations between factors studied. P-values from Bivariate analyses were adjusted by False Discovery Rate (FDR). Factors significantly associated with best neutralization score in the Bivariate analyses, with q-values < 0.1 were selected for further Multivariable modeling.

Kaplan-Meier survival analyses with Log rank test were performed to look at the relationship between time post-infection when certain level of neutralization was achieved and different levels of clinical parameters. Two different versions of this analysis were performed either including all the participants described in this study (n = 439) or including only the individuals having reached the level of neutralization assessed at some point during the study. P-values less than 0.05 were considered statistically significant.

### Neutralization assays

Plasma collected from the Protocol C cohort eligible participants were heat-inactivated at 56° C for 45min prior to use in neutralization assays.

Briefly, WT and mutant pseudoviruses were generated by co-transfection of 293T cells with an Env-expressing plasmid and an Env-deficient genomic backbone plasmid (pSG3ΔEnv), as described previously [86]. Pseudoviruses were harvested 72h post transfection for use in neutralization assays. Neutralizing activity was assessed in absence of DEAE-dextran using single-round replication in TZM-bl target cells by measuring luciferase activity after 72h. Pseudoviruses incorporating single amino acid mutations were generated by Quickchange mutagenesis (Stratagene). Kifunensine-treated pseudoviruses were produced by treating 293T cells with 25 μM kifunensine (α-mannosidases inhibitor, preventing the trimming of Man8/9 glycan to Man5) on the day of transfection [87]. Chimeric HIV-2 clones containing the partial or full length MPER of HIV-1 were derived from the parental HIV-2 7312A clone in which the HIV-2 Env MPER sequence (QKLN- SWDVFGNWFDLASWVKYIQ) was replaced by the complete (HIV-2 C1), the N-terminal segment (HIV-2 C3, 2F5 epitope) or the C-terminal segment (HIV-2 C4, 4E10/10E8 epitopes) of HIV-1 YU2 MPER sequence LALDKWASLWNWFDITKWLWYIK, as described [14]. To determine ID50 values, serial dilutions of plasma were incubated with virus and the dose-response curves were fitted using nonlinear regression. For competition assays, plasma dilutions were pre-incubated 30 minutes at room temperature with 25μg/mL of gp120 cores (RCS3/KO-RSC3, TriMut/KO-TriMut) or 10μg/mL of MPER peptide. Both KO cores bore the combined D368R+E370A+D474A mutations essentially affecting binding of CD4bs-specific monoclonal bnAbs. An effect of a particular mutation, competitor or virus treatment on was called positive when it resulted in a > 2-fold or >20% decrease in neutralization of ID50 compared to WT virus, KO-competitor or untreated virus.

Neutralization score on the 37-virus panel (37v-panel) was calculated using the same formula used for the 6-virus panel (6v-panel) as detailed above.

### Recombinant Envelope glycoproteins

HIV-1 MN ENVgp41 (E.Coli, #12027) and HIV-1 IIIB GAGp24 (Baculo, #12028) recombinant protein were obtained through the NIH AIDS Reagent Program, Division of AIDS, NIAID) (DAIDS Immunodiagnostics, Inc).

All gp120 monomers and core gp120 proteins were expressed by transfecting 293F cells in plasma-free medium (OptiMEM, Invitrogen, Carlsbad, CA). In brief, cell culture supernatants of 293-transfected cells were harvested 4 days post-transfection, cleared, filtered and two protease inhibitor tablets (Roche) per liter of supernatant were added to limit proteolysis. Gp120 proteins were purified on Galanthus nivalis lectin-bound agarose columns (Vector Laboratories). The columns were then washed sequentially with 10 column volumes of phosphate-buffered saline (PBS) (pH 7.4) containing 0.5 M NaCl, followed by 10 column volumes of PBS (pH 7.4). The lectin-bound glycoproteins were eluted with a total of 10 column volumes of elution buffer (PBS buffer [pH 7.4] with 0.5 M methyl-D-mannopyranoside and 10mM imidazole). The mannoside-eluted glycoproteins were pooled, dialyzed against phosphate-buffered saline (PBS) pH 7.4 before being size excluded on a Superose 6. Fractions containing monomers were concentrated with Amicon Ultra 30,000 MWCO centrifugal filter devices (Millipore, Bedford, MA). Finally, the purified proteins were subjected to sodium dodecyl sulfate-polyacrylamide gel electrophoresis and ELISA analysis, and protein purity was verified.

### Plasma adsorptions

Plasma adsorptions with gp120-coupled beads were performed using tosyl-activated magnetic beads, as described previously [88]. Bead coupling was performed at a ratio of 1mg gp120 of a single strain per 25mg of beads. Three to four rounds of adsorption were performed to ensure complete removal of antigen-specific antibodies as verified by ELISA. Gp120 from multiple strains were used individually in independent experiments (S3 Table in S1 Text) and chosen based on an ENV pseudotyped virus neutralization by a given plasma sample. For plasma adsorptions performed in the presence of b6, gp120- coupled beads were pre-incubated with 500 μg/ml IgG b6 for 30min at room temperature before adding plasma. The mAb b6 was procured by the IAVI Neutralizing Antibody Consortium. An effect of gp120 absorption was called positive when it resulted in a >2 fold decrease in neutralization of ID50 compared to the untreated plasma.

### ELISA assays

Half-area 96-well ELISA plates were coated overnight at 4C with 50 μL PBS containing 50 to 250 ng of RCS3, KO-RSC3, ENV-gp120, ENV-gp41, GAG-p24 or anti-human IgG Fc per well. The wells were washed four times with PBS containing 0.05% Tween 20 and blocked with 3% BSA at room temperature for 1 h. Serial dilutions of plasma were then added to the wells, and the plates were incubated at room temperature for 1 hour. After washing four times, goat anti-human IgG F(ab’)2 conjugated to alkaline phosphatase (Pierce), diluted 1:1000 in PBS containing 1% BSA and 0.025% Tween 20, was added to the wells. The plates were incubated at room temperature for 1 h, washed four times, and developed by adding alkaline phosphatase substrate (Sigma) diluted in alkaline phosphatase staining buffer (pH 9.8), according to the manufacturer’s instructions. For avidity assessment, washes were performed in presence of 1.5M NaSCN or 3M NaSCN. Optical density at 405 nm was read on a microplate reader (Molecular Devices). Endpoint titers of the plasma antibodies were defined as the last reciprocal plasma dilution at which the background-corrected OD signal was greater than or equal to 0.1 and EC50 values were calculated using Prism6 (GraphPad).

## Supporting Information

S1 Text Supporting figures and tables. Supporting S1-S12 Figures and S1-S4 Tables with corresponding legends
(PDF)
