# Supplementary material for: Broadly Neutralizing Antibody Responses in a Large Longitudinal Sub-Saharan HIV Primary Infection Cohort
Source: PLoS Pathog. 2016 Jan 14;12(1):e1005369. doi: 10.1371/journal.ppat.1005369 (PMC4713061; doi:10.1371/journal.ppat.1005369)
Supplement: S1 Text — Supporting S1-S12 Figures and S1-S4 Tables with corresponding legends (PDF) [file ppat.1005369.s001.pdf]

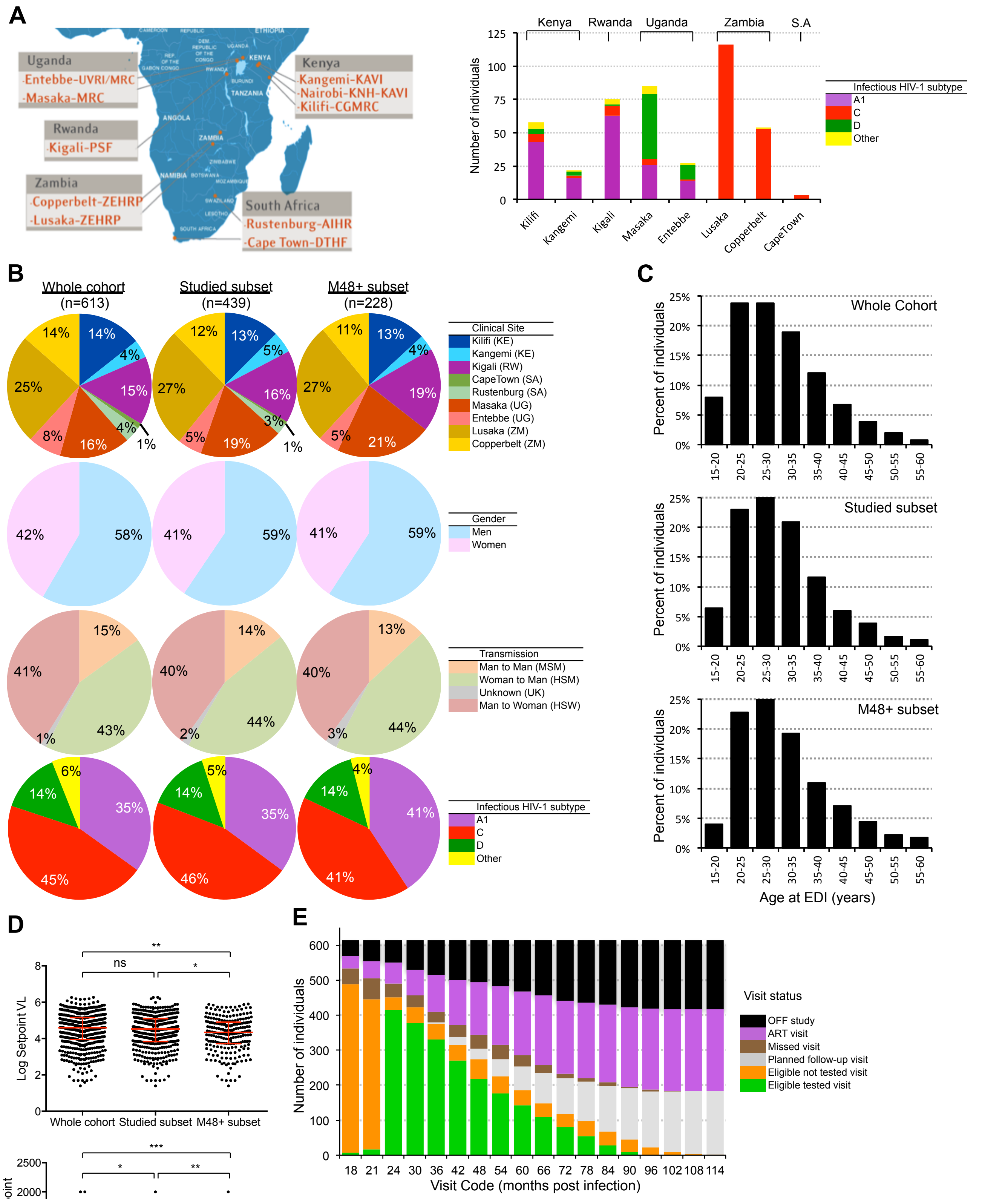

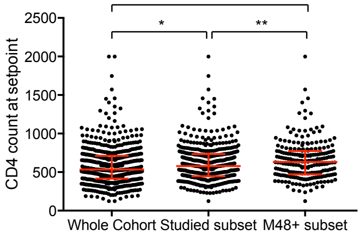

S1 Figure: Protocol C cohort. (A) Map and HIV-subtype prevalence of the IAVI-sponsored Clinical Research Centers (CRC) participating in the Protocol C program. HIV-1 "Other" includes A1A2, A1C, A1CD, A1D, CK, CRF02_AG, CRF11_CPX, and BC. S.A. : South Africa. (B, C, D) Comparison of participants included in this study (N=439, M48+ subset: N=228) to the entire Protocol C cohort (N=613) for various demographic (B, C) and clinical parameters (D). Mann Whitney t-test significance and median with Interquartile are indicated. (E) Status of all longitudinal Protocol C donors regarding 6-virus panel neutralization screening. ART: Anti-Retroviral Therapy.

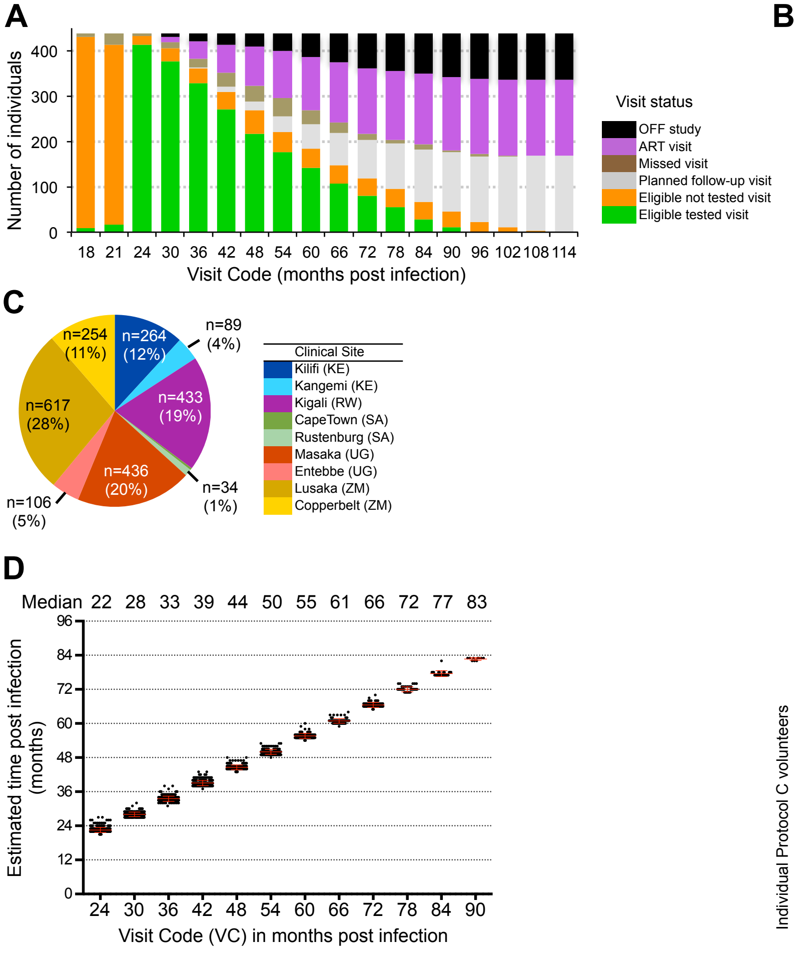

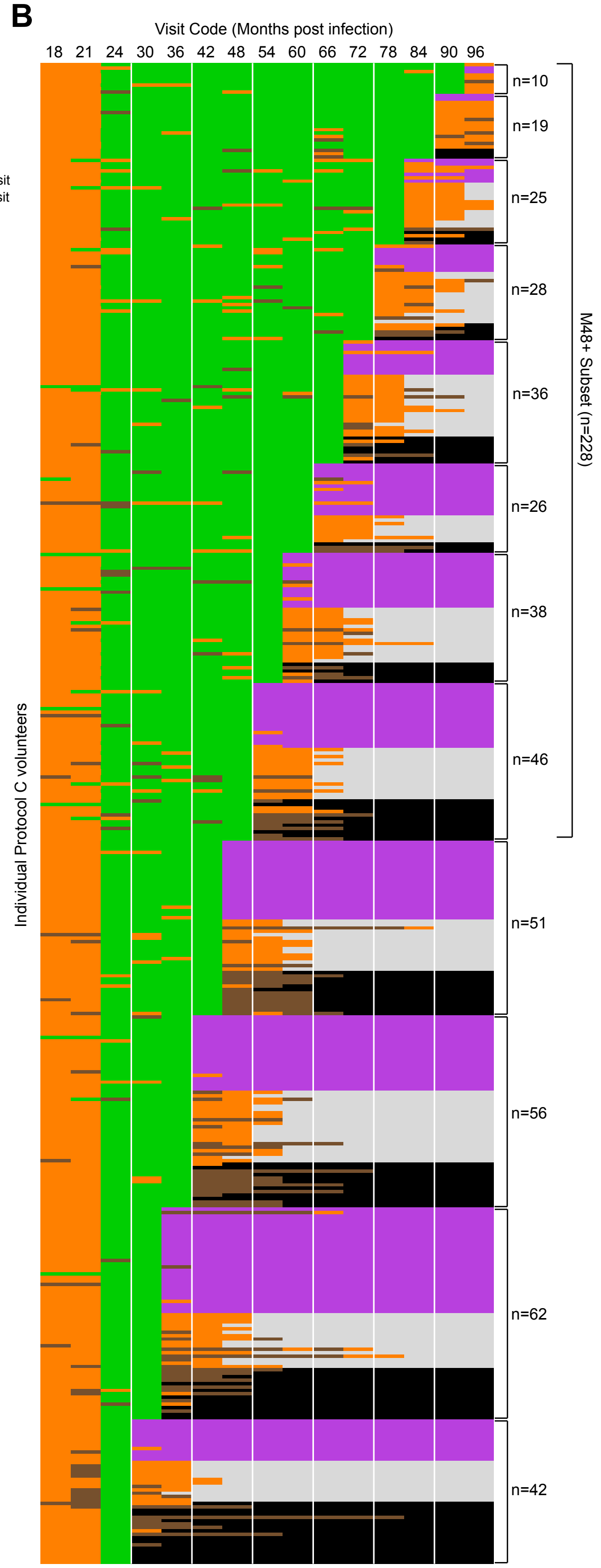

S2 Figure: Detailed follow-up of Protocol C participants included in this study.

(A,B) Sample status regarding 6-virus panel neutralization screening, detailed (A) by time point or by individuals (B) organized by time of follow-up. (C) Clinical site of origin of neutralization-tested samples. (D) Variation of visit code (VC) values from the calculated number of months between the visit date and the estimated date of infection.

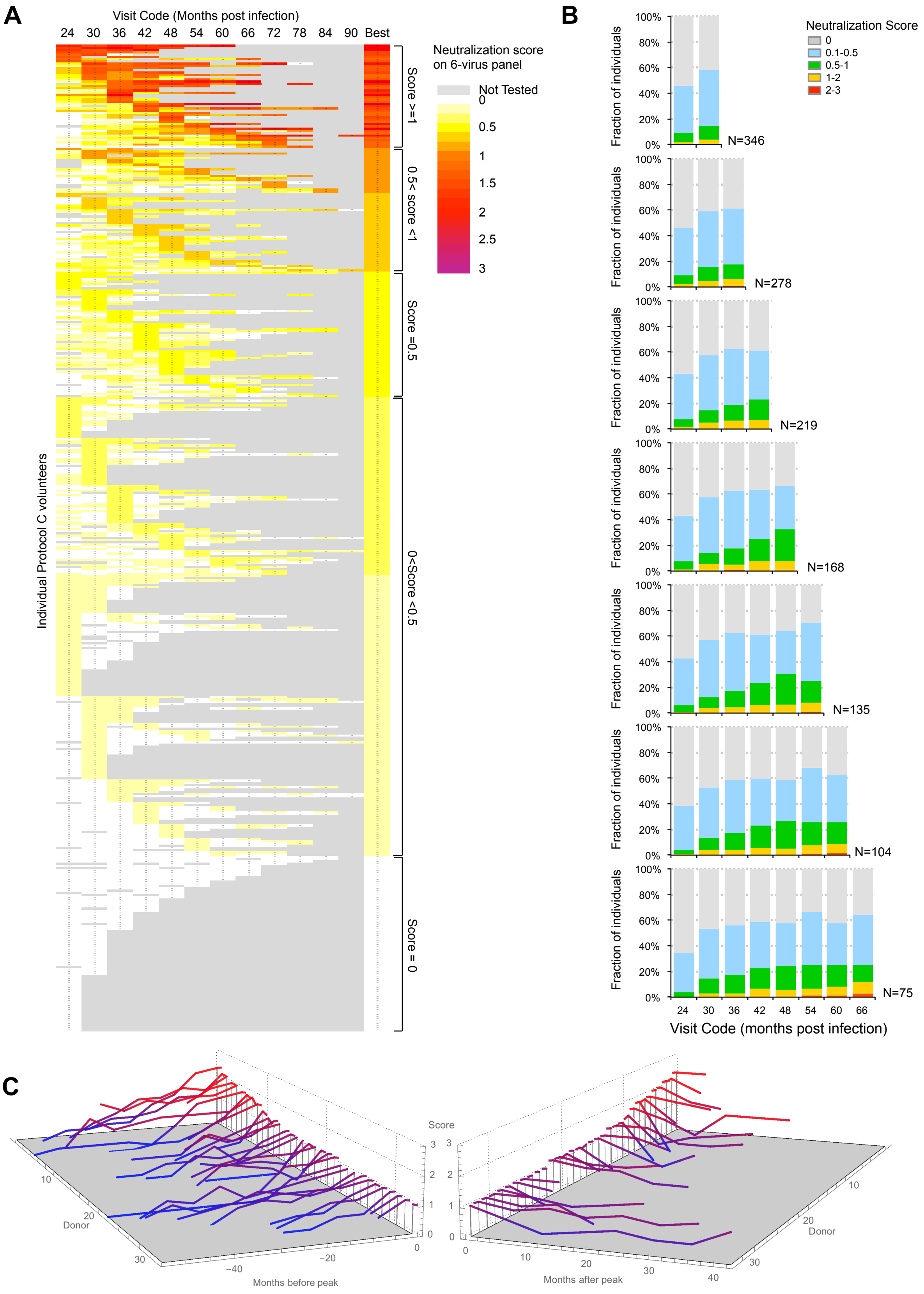

S3 Figure: Evolution of broadly neutralizing activity for Protocol C participants.
(A) Neutralization score on the 6-virus panel of plasma samples collected at different visits from individual Protocol C participants. Best neutralization score category and time post infection to reach this score category were respectively used as primary and secondary donor ranking parameters. (B) Longitudinal 6-virus panel neutralization score of plasma samples from Protocol C participants tested for the same visits. The number of participants included is indicated. (C) Detailed evolution of neutralization score over time (months) before and after neutralization peak for individual Protocol C best neutralizers, organized by best neutralization score across the study period.

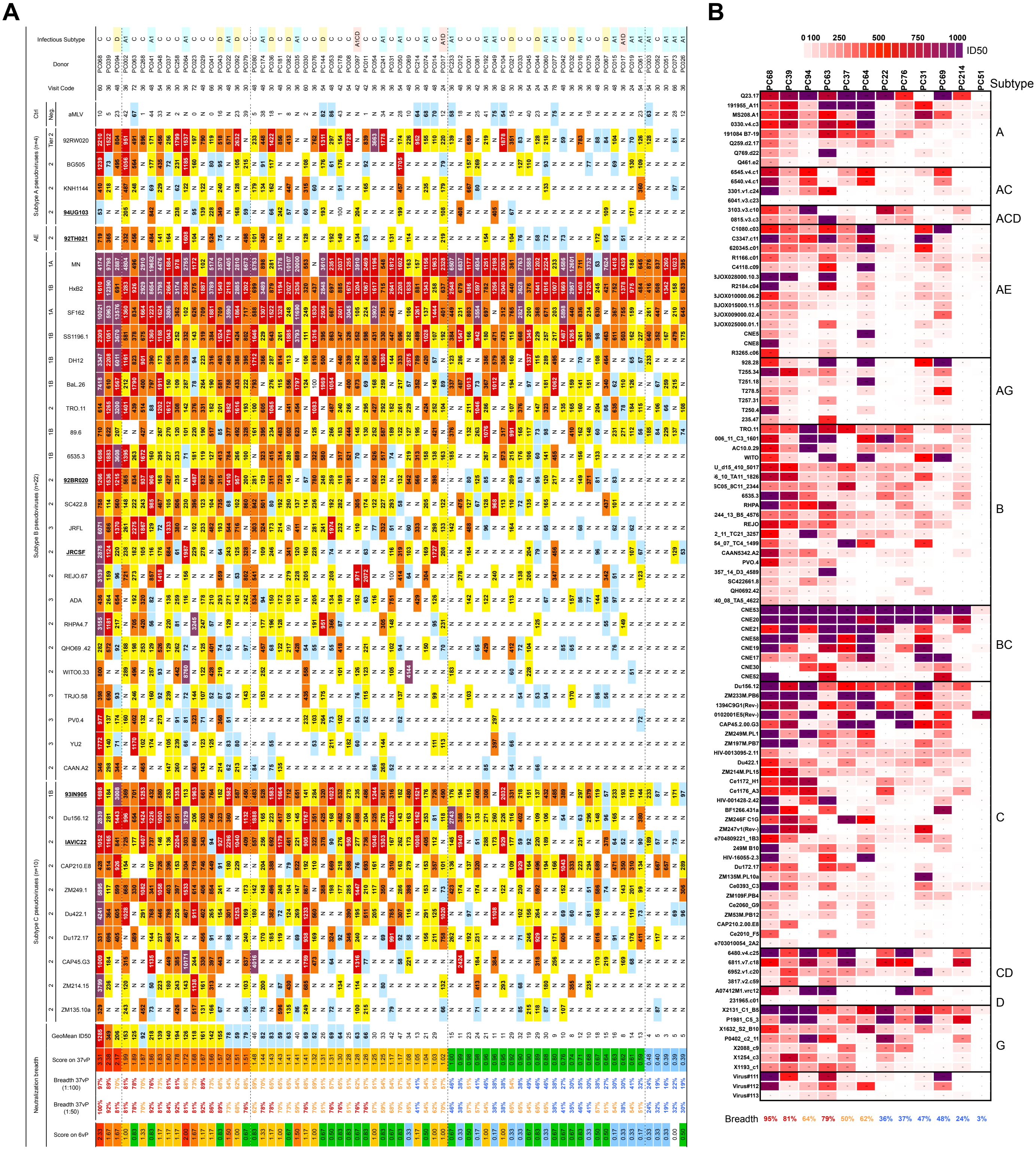

S4 Figure: Neutralization breadth on larger virus panels and correlation with neutralization score on 6-virus panel.
Plasma samples from Protocol C participants (Table S2) were tested for neutralization against a medium panel (N=37) (A) and a large reference panel (N=105, Seaman 2010) (B) of cross-clade tier-1-3 pseudoviruses. (A) Neutralization ID50 values on the 37-virus panel (37vP) are color-coded as follows: (blue) 50 ≤ ID50 <100; (yellow) 100 ≤ ID50 <300; (orange) 300≤ ID50 <900; (red) 900≤ ID50 <2700; (purple) ID50 ≥2700. Neutralization breadth is given as the percentage of viruses neutralized at 1:50 or 1:100 plasma dilution and color-coded as follows: (blue) 0-50% viruses neutralized; (yellow) 50-75% viruses neutralized; (red) >75% viruses neutralized. Neutralization scores were calculated using the algorithm given in the Methods section for the 6-virus panel and are color-coded as follows: (blue) 0<score<0.5; (green) 0.5≤ score <1; (yellow) 1≤ score <1.5; (orange) 1.5≤ score <2 and (red) 2≤ score <3. (B) Neutralization breadth on the 105-virus panel (105vP) is given as the percentage of viruses neutralized at 1:50 plasma dilution and color-coded as in (A)

C

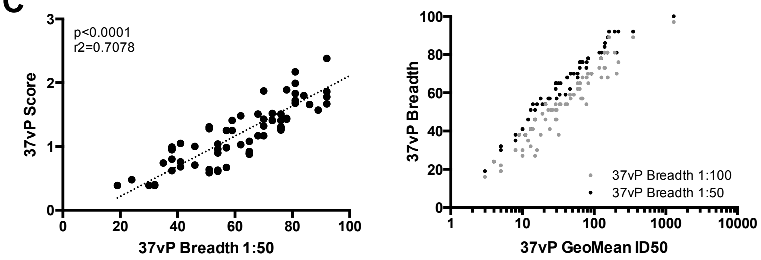

D

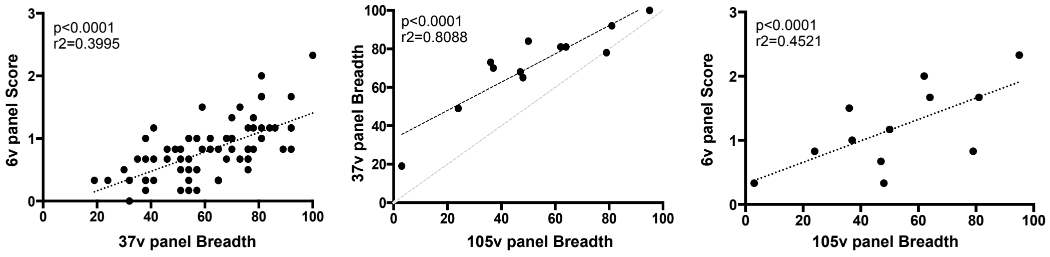

S4 Figure: Neutralization breadth on larger virus panels and correlation with neutralization score on 6-virus panel.
(C) Linear regression analyses between the neutralization breadth calculated on the 37v-panel using at 1:50 or 1:100 plasma dilution ID50 cut-off and the neutralization potency on 37v-panel (GeoMean of ID50). The p-value and R-square value are indicated. (D) Neutralization breadth was calculated as the percentage of viruses neutralized at 50% at 1:50 plasma dilution. Neutralization score on the 6v-panel was calculated as indicated in the Methods section. Linear regression (black dotted line) analyses between the neutralization breadth and score calculated on the 3 different virus panels are plotted. The p-values and R-square values are indicated. The theoretical perfect linear correlation between breadths on the 37v- and 105v-panel is shown as a grey dotted line for reference.

A

Bivariate Generalized Linear Modeling							
Variables	N	EstCoef.	p-value	q-value	ExpEst	L95	U95
**Time of Follow-up All**	439	0.13	6.0E-15	**2.5E-13**	1.14	1.10	1.18
**Time of Follow-up M48+**	228	0.02	0.47	1.00	1.02	0.97	1.08

B

Bivariate Generalized Linear Modeling								
Variables		N	EstCoef	p-value	q-value	ExpEst	L95	U95
**Time of Follow-up**		228	0.02	0.47	1.00	1.02	0.97	1.08
**Gender**	Women vs Men	93 / 135	0.05	0.66	1.00	1.05	0.84	1.32
**Age_EDI**		228	0.00	0.61	1.00	1.00	0.99	1.02
**Risk**	DC vs MSM	169 / 30	-0.22	0.18	1.00	0.80	0.58	1.12
	DC vs OHS	169 / 23	-0.38	0.040	1.00	0.68	0.48	1.00
	DC vs UK	169 / 6	-0.44	0.21	1.00	0.64	0.34	1.39
**Transmission**	HSM vs HSW	101 / 91	-0.09	0.47	1.00	0.92	0.72	1.16
	HSM vs MSM	101 / 30	-0.23	0.20	1.00	0.80	0.57	1.14
	HSM vs UK	101 / 6	-0.45	0.21	1.00	0.64	0.34	1.39
**Subtype**	A1 vs C	93 / 94	0.40	0.0017	**0.071**	1.49	1.16	1.90
	A1 vs D	93 / 32	0.16	0.35	1.00	1.18	0.84	1.67
	A1 vs Other	93 / 9	0.27	0.37	1.00	1.31	0.76	2.46
**Recruitment Site**	Kigali vs Kilifi	43 / 30	-0.20	0.31	1.00	0.82	0.55	1.22
	Kigali vs Kangemi	43 / 8	0.20	0.54	1.00	1.22	0.67	2.44
	Kigali vs Masaka	43 / 49	-0.04	0.83	1.00	0.96	0.68	1.36
	Kigali vs Entebbe	43 / 11	-0.50	0.083	1.00	0.6	0.35	1.1
	Kigali vs Lusaka	43 / 62	0.32	0.064	1.00	1.38	0.99	1.92
	Kigali vs Copperbelt	43 / 25	0.33	0.13	1.00	1.38	0.92	2.12
	Kigali vs Cape Town	43 / 0						
**LogVL_Setpoint**		227	0.31	2.3E-07	**9.7E-06**	1.37	1.21	1.55
**CD4_Setpoint**		226	0.00	0.052	1.00	1.00	1.00	1.00

C

Bivariate Generalized Linear Modeling								
Variables		N	EstCoef	p-value	q-value	ExpEst	L95	U95
**HLA_A*01**	1 vs. 0	30 / 198	0.01	0.97	1.00	1.01	0.73	1.42
**HLA_A*02**	1 vs. 0	79 / 149	0.17	0.16	1.00	1.18	0.94	1.50
**HLA_A*03**	1 vs. 0	23 / 205	-0.57	0.0022	**0.094**	0.57	0.40	0.83
**HLA_A*23**	1 vs. 0	33 / 195	0.07	0.68	1.00	1.07	0.79	1.48
**HLA_A*26**	1 vs. 0	6 / 222	-0.38	0.28	1.00	0.68	0.37	1.48
**HLA_A*29**	1 vs. 0	18 / 210	0.03	0.89	1.00	1.03	0.69	1.61
**HLA_A*30**	1 vs. 0	76 / 152	-0.03	0.82	1.00	0.97	0.77	1.24
**HLA_A*33**	1 vs. 0	16 / 212	0.15	0.49	1.00	1.16	0.77	1.85
**HLA_A*34**	1 vs. 0	19 / 209	-0.19	0.34	1.00	0.82	0.56	1.26
**HLA_A*36**	1 vs. 0	20 / 208	-0.21	0.30	1.00	0.81	0.56	1.23
**HLA_A*66**	1 vs. 0	10 / 218	-0.47	0.090	1.00	0.62	0.38	1.12
**HLA_A*68**	1 vs. 0	55 / 173	0.06	0.66	1.00	1.06	0.82	1.38
**HLA_A*74**	1 vs. 0	29 / 199	-0.11	0.52	1.00	0.90	0.65	1.27
**HLA_B*07**	1 vs. 0	35 / 193	-0.11	0.47	1.00	0.89	0.66	1.23
**HLA_B*08**	1 vs. 0	9 / 219	0.44	0.13	1.00	1.55	0.92	2.89
**HLA_B*14**	1 vs. 0	27 / 201	0.06	0.76	1.00	1.06	0.76	1.52
**HLA_B*15**	1 vs. 0	74 / 154	-0.04	0.71	1.00	0.96	0.76	1.22
**HLA_B*18**	1 vs. 0	9 / 219	0.31	0.29	1.00	1.36	0.80	2.54
**HLA_B*35**	1 vs. 0	9 / 219	-0.09	0.76	1.00	0.91	0.54	1.71
**HLA_B*42**	1 vs. 0	27 / 201	0.00	1.00	1.00	1.00	0.72	1.43
**HLA_B*44**	1 vs. 0	29 / 199	-0.03	0.86	1.00	0.97	0.70	1.37
**HLA_B*45**	1 vs. 0	26 / 202	0.24	0.18	1.00	1.27	0.91	1.83
**HLA_B*49**	1 vs. 0	18 / 210	-0.27	0.20	1.00	0.76	0.52	1.18
**HLA_B*51**	1 vs. 0	9 / 219	0.33	0.25	1.00	1.40	0.83	2.62
**HLA_B*53**	1 vs. 0	44 / 184	0.07	0.61	1.00	1.08	0.82	1.44
**HLA_B*57**	1 vs. 0	27 / 201	-0.16	0.38	1.00	0.86	0.61	1.23
**HLA_B*58**	1 vs. 0	58 / 170	0.05	0.68	1.00	1.05	0.82	1.37
**HLA_B*81**	1 vs. 0	12 / 216	-0.17	0.49	1.00	0.84	0.53	1.43
**HLA_C*02**	1 vs. 0	48 / 180	0.08	0.55	1.00	1.09	0.83	1.44
**HLA_C*03**	1 vs. 0	30 / 198	0.27	0.11	1.00	1.31	0.95	1.85
**HLA_C*04**	1 vs. 0	74 / 154	0.08	0.50	1.00	1.08	0.86	1.38
**HLA_C*06**	1 vs. 0	61 / 167	-0.16	0.19	1.00	0.85	0.67	1.09
**HLA_C*07**	1 vs. 0	84 / 144	-0.07	0.55	1.00	0.93	0.74	1.18
**HLA_C*08**	1 vs. 0	33 / 195	-0.12	0.47	1.00	0.89	0.65	1.24
**HLA_C*16**	1 vs. 0	28 / 200	0.09	0.62	1.00	1.09	0.79	1.55
**HLA_C*17**	1 vs. 0	30 / 198	0.01	0.97	1.00	1.01	0.73	1.42
**HLA_C*18**	1 vs. 0	21 / 207	0.01	0.94	1.00	1.01	0.70	1.52
**HLA_DRB1*01**	1 vs. 0	39 / 188	0.04	0.81	1.00	1.04	0.78	1.41
**HLA_DRB1*03**	1 vs. 0	76 / 151	0.01	0.91	1.00	1.01	0.80	1.29
**HLA_DRB1*04**	1 vs. 0	6 / 221	-0.05	0.88	1.00	0.95	0.51	2.06
**HLA_DRB1*07**	1 vs. 0	32 / 195	-0.06	0.71	1.00	0.94	0.69	1.31
**HLA_DRB1*08**	1 vs. 0	21 / 206	0.33	0.090	1.00	1.39	0.97	2.07
**HLA_DRB1*09**	1 vs. 0	8 / 219	-0.68	0.030	1.00	0.51	0.29	0.98
**HLA_DRB1*10**	1 vs. 0	12 / 215	0.08	0.74	1.00	1.09	0.69	1.85
**HLA_DRB1*11**	1 vs. 0	75 / 152	0.13	0.30	1.00	1.13	0.90	1.44
**HLA_DRB1*12**	1 vs. 0	17 / 210	-0.29	0.18	1.00	0.75	0.51	1.17
**HLA_DRB1*13**	1 vs. 0	73 / 154	-0.15	0.22	1.00	0.86	0.68	1.10
**HLA_DRB1*15**	1 vs. 0	60 / 167	0.18	0.16	1.00	1.20	0.94	1.55
**HLA_DQB1*02**	1 vs. 0	70 / 157	0.00	0.99	1.00	1.00	0.79	1.28
**HLA_DQB1*03**	1 vs. 0	75 / 152	-0.02	0.88	1.00	0.98	0.78	1.25
**HLA_DQB1*04**	1 vs. 0	41 / 186	0.05	0.72	1.00	1.05	0.79	1.43
**HLA_DQB1*05**	1 vs. 0	79 / 148	0.01	0.90	1.00	1.01	0.81	1.29
**HLA_DQB1*06**	1 vs. 0	133 / 94	0.05	0.66	1.00	1.05	0.84	1.32
**KIR_2DL1**	1 vs. 0	207 / 12	0.04	0.86	1.00	1.05	0.61	1.65
**KIR_2DL2**	1 vs. 0	142 / 78	0.14	0.23	1.00	1.16	0.91	1.46
**KIR_2DL3**	1 vs. 0	201 / 23	-0.38	0.030	1.00	0.68	0.47	0.96
**KIR_2DL4**	1 vs. 0	223 / 2	-0.24	0.69	1.00	0.78	0.18	2.13
**KIR_2DL5**	1 vs. 0	132 / 87	0.19	0.12	1.00	1.21	0.95	1.53
KIR_2DL5a	1 vs. 0	20 / 201	0.29	0.15	1.00	1.34	0.92	2.04
KIR_2DL5b	1 vs. 0	121 / 99	0.15	0.19	1.00	1.17	0.93	1.47
**KIR_2DP1**	1 vs. 0	220 / 5	0.12	0.76	1.00	1.13	0.47	2.22
**KIR_2DS1**	1 vs. 0	63 / 162	0.11	0.40	1.00	1.11	0.87	1.43
**KIR_2DS2**	1 vs. 0	105 / 118	0.04	0.75	1.00	1.04	0.83	1.30
**KIR_2DS3**	1 vs. 0	55 / 168	-0.26	0.050	1.00	0.77	0.60	1.00
**KIR_2DS4**	1 vs. 0	210 / 11	-0.30	0.25	1.00	0.74	0.42	1.19
KIR_2DS4_1	1 vs. 0	165 / 58	0.00	0.99	1.00	1.00	0.77	1.29
KIR_2DS4_2	1 vs. 0	121 / 98	-0.04	0.74	1.00	0.96	0.77	1.21
**KIR_2DS5**	1 vs. 0	104 / 120	0.27	0.020	0.91	1.31	1.04	1.65
**KIR_3DL1**	1 vs. 0	221 / 2	-0.24	0.69	1.00	0.79	0.18	2.14
**KIR_3DP1**	1 vs. 0	210 / 3	0.06	0.91	1.00	1.06	0.33	2.46
KIR_3DP1_3	1 vs. 0	210 / 3	0.06	0.91	1.00	1.06	0.33	2.46
KIR_3DP1_124	1 vs. 0	30 / 188	-0.13	0.43	1.00	0.88	0.64	1.23
**KIR_3DS1**	1 vs. 0	40 / 185	0.23	0.13	1.00	1.26	0.95	1.70

D

Bivariate Generalized Linear Modeling						
Variables	EstCoef	p-value	q-value	ExpEst	L95	U95
**LOGVL_SP**	0.31	2.3E-07	**1.5E-06**	1.37	1.21	1.55
**LOGVL_M06**	0.47	2.8E-13	**1.8E-12**	1.59	1.41	1.80
**LOGVL_M09**	0.48	1.7E-16	**1.1E-15**	1.62	1.45	1.81
**LOGVL_M12**	0.49	3.2E-16	**2.1E-15**	1.64	1.46	1.84
**LOGVL_M15**	0.49	8.9E-12	**5.8E-11**	1.63	1.40	1.89
**LOGVL_M18**	0.50	4.5E-15	**3.0E-14**	1.65	1.46	1.86
**LOGVL_M21**	0.47	1.9E-12	**1.2E-11**	1.60	1.39	1.83
**LOGVL_M24**	0.41	5.3E-11	**3.4E-10**	1.51	1.32	1.72
**LOGVL_M30**	0.42	7.9E-12	**5.1E-11**	1.53	1.35	1.73
**LOGVL_M36**	0.44	1.0E-11	**6.7E-11**	1.55	1.35	1.76
**LOGVL_M42**	0.44	1.5E-12	**9.9E-12**	1.56	1.37	1.76
**LOGVL_M48**	0.39	1.9E-09	**1.2E-08**	1.48	1.30	1.69
**LOGVL_AUC**	0.02	2.8E-19	**1.9E-18**	1.01	1.01	1.02

Bivariate Generalized Linear Modeling						
Variables	EstCoef	p-value	q-value	ExpEst	L95	U95
**CD4_SP**	-0.00	0.052	0.34	1.00	1.00	1.00
**CD4_M06**	-0.00	0.031	0.44	1.00	1.00	1.00
**CD4_M09**	-0.00	2.1E-05	**2.8E-04**	1.00	1.00	1.00
**CD4_M12**	-0.00	2.9E-04	**3.8E-03**	1.00	1.00	1.00
**CD4_M15**	-0.00	4.2E-07	**5.5E-06**	1.00	1.00	1.00
**CD4_M18**	-0.00	4.7E-06	**6.1E-05**	1.00	1.00	1.00
**CD4_M21**	-0.00	1.5E-04	**2.0E-03**	1.00	1.00	1.00
**CD4_M24**	-0.00	2.0E-04	**2.6E-03**	1.00	1.00	1.00
**CD4_M30**	-0.00	0.0015	**0.019**	1.00	1.00	1.00
**CD4_M36**	-0.00	2.4E-04	**3.2E-03**	1.00	1.00	1.00
**CD4_M42**	-0.00	0.0021	**0.028**	1.00	1.00	1.00
**CD4_M48**	-0.00	0.0087	0.11	1.00	1.00	1.00
**CD4_AUC**	-0.00	4.0E-04	**5.2E-03**	1.00	1.00	1.00

S5 Figure: Regression analysis of the association between best neutralization score and clinical parameters.

(A) Bivariate GLM correlation analysis between last time point tested (Time of Follow-up, in months post infection) and the best neutralization score for the Protocol C participants of either the studied subset (N=439) or the M48+ subset (N=228). (B,C,D) Bivariate GLM correlation analyses of the listed variables with the best neutralization score for the M48+ subset of Protocol C participants. Number of participants in each subgroup (N) is indicated. DC: Discordant couple, OHS: Other Heterosexual transmission, HSM: Women to Men Heterosexual transmission, HSW: Men to Women Heterosexual transmission, MSM: Men who have Sex with Men. Log10_VL_X: Log10 viral load at a given visit in months post infection; AUC: area under the curve for Log10 viral load between 6 and 48 months post infection. Estimated coefficients (EstCoef), p-values, q-values, odd ratios (ExpEst) upper (U95) and lower (L95) values of the 95% confidence interval are indicated. P-values are color coded as follows: 0.01< p-value < 0.05, in green; 0.001< p-value < 0.01, in yellow; 2E-16 < p-value <0.001, in red. Q-values below 0.1 are indicated in bold. The number (N) of participants in each subgroup is indicated.

A

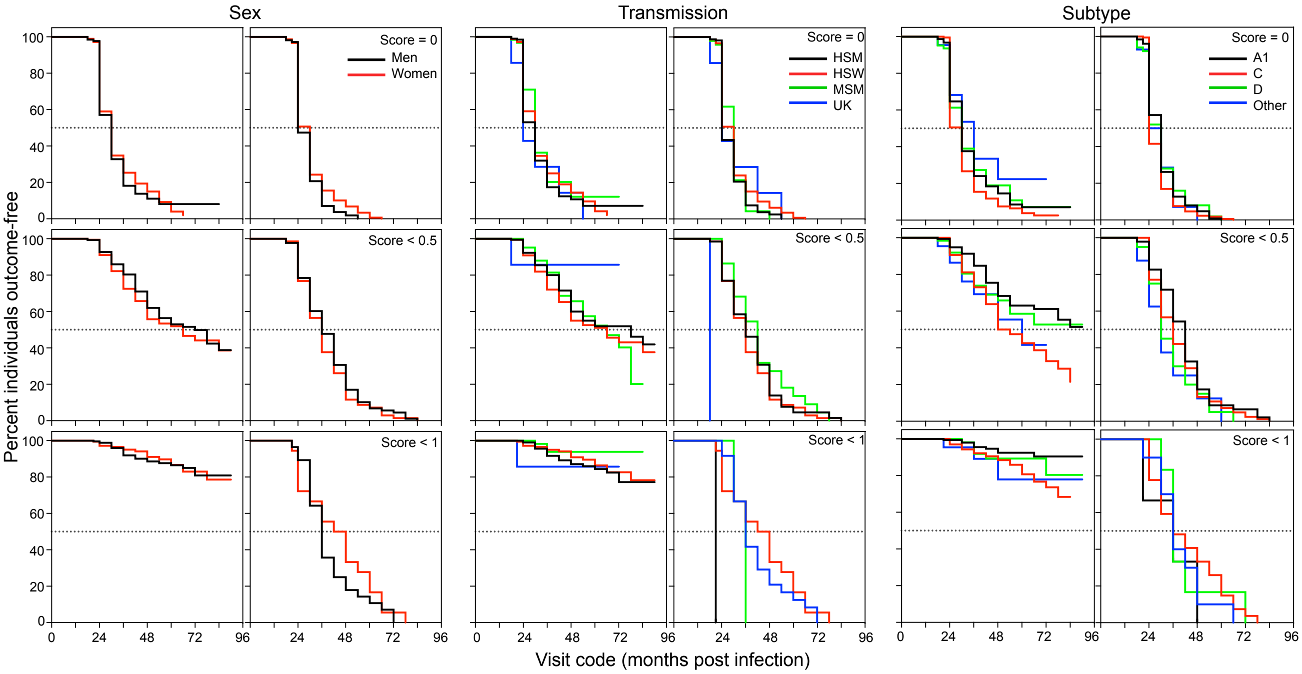

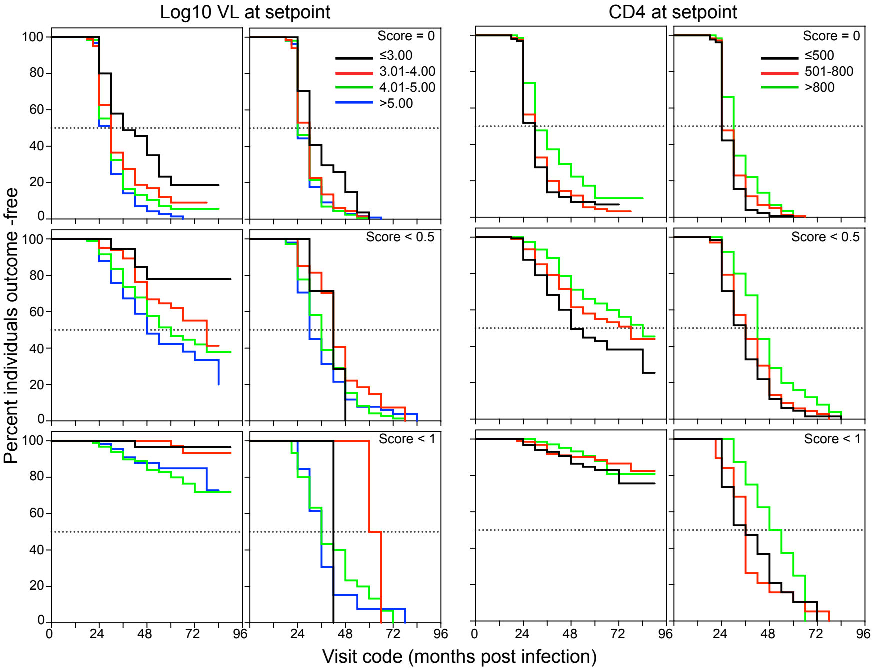

Number of events		All	Subset score criteria		
Variables		/	>0	≥0.5	≥1
**Sex**	Men	213	213	88	28
	Women	148	148	69	18
**Transmission**	HSM	161	161	65	24
	HSW	146	146	69	18
	MSM	47	47	22	3
	UK	7	7	1	1
**Subtype**	A1	126	126	46	10
	C	171	171	83	27
	D	50	50	20	6
	Other	14	14	8	3
**Viral load**	<=3.00	27	27	7	1
	3.01-4.00	66	66	27	2
	4.01-5.00	158	158	72	30
	>5.00	108	108	51	13
**CD4**	<500	128	128	64	19
	500-800	174	174	68	19
	<800	59	59	25	8

B

Log-Rank Test			
	Time to Score		
**Variables**	**>0**	**≥0.5**	**≥1**
**Subtype**	0.25	0.29	0.76
**Gender**	0.037	0.40	0.37
**Risk**	0.98	0.70	0.35
**Transmission**	0.21	0.84	0.43
**Age_Group**	0.67	0.67	0.65
**Viral_Load**	0.018	0.21	0.65
**CD4**	0.0022	0.019	0.46

S6 Figure: Kinetic of development of neutralization score and clinical parameters.
(A) Kaplan Meier curves recording the time for Protocol C participants within the indicated subgroups to reach a neutralization score >0, ≥0.5 or ≥1 during the follow-up time post infection, including all individuals selected in this study (left) or only the few individuals reaching the assessed neutralization score at some point during the study (right). The number of participants included in each subgroup is indicated in the lower right table. (B) Log-Rank test p-values for the analyses restricted to criteria-positive individuals (Right panels from (A)).

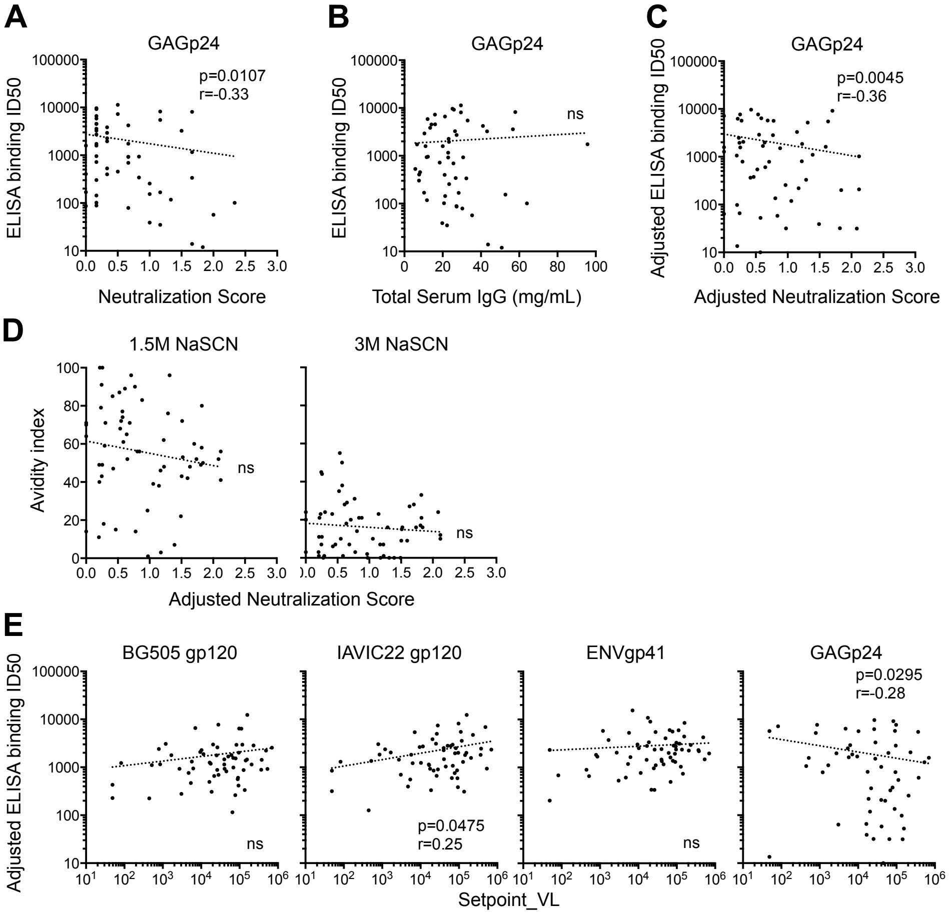

S7 Figure: Correlation between plasma IgG titers and development of broadly neutralizing antibody responses in Protocol C. Plasma samples from Protocol C participants (M24-72, mean= 36.6mpi), were tested by ELISA for total IgG titers, and IgG binding activity to recombinant BG505 gp120 (subtype A), IAVIC22 gp120 (Subtype B), MN gp41 (Subtype B) and IIIB GAGp24 (Subtype B). Avidity index for IAVIC22-gp120 IgG titers were calculated from high salt (1.5M or 3M NaSCN) ELISA experiments. ELISA binding ID50 and participants' best neutralization score on the 6v-panel were standardized to a reference concentration of 20mg/mL of total plasma IgG. Linear, semi-Log or Log-log regressions are shown as dotted lines, and Spearman correlation analyses results (p-values and r-values) are indicated; (ns) not significant.

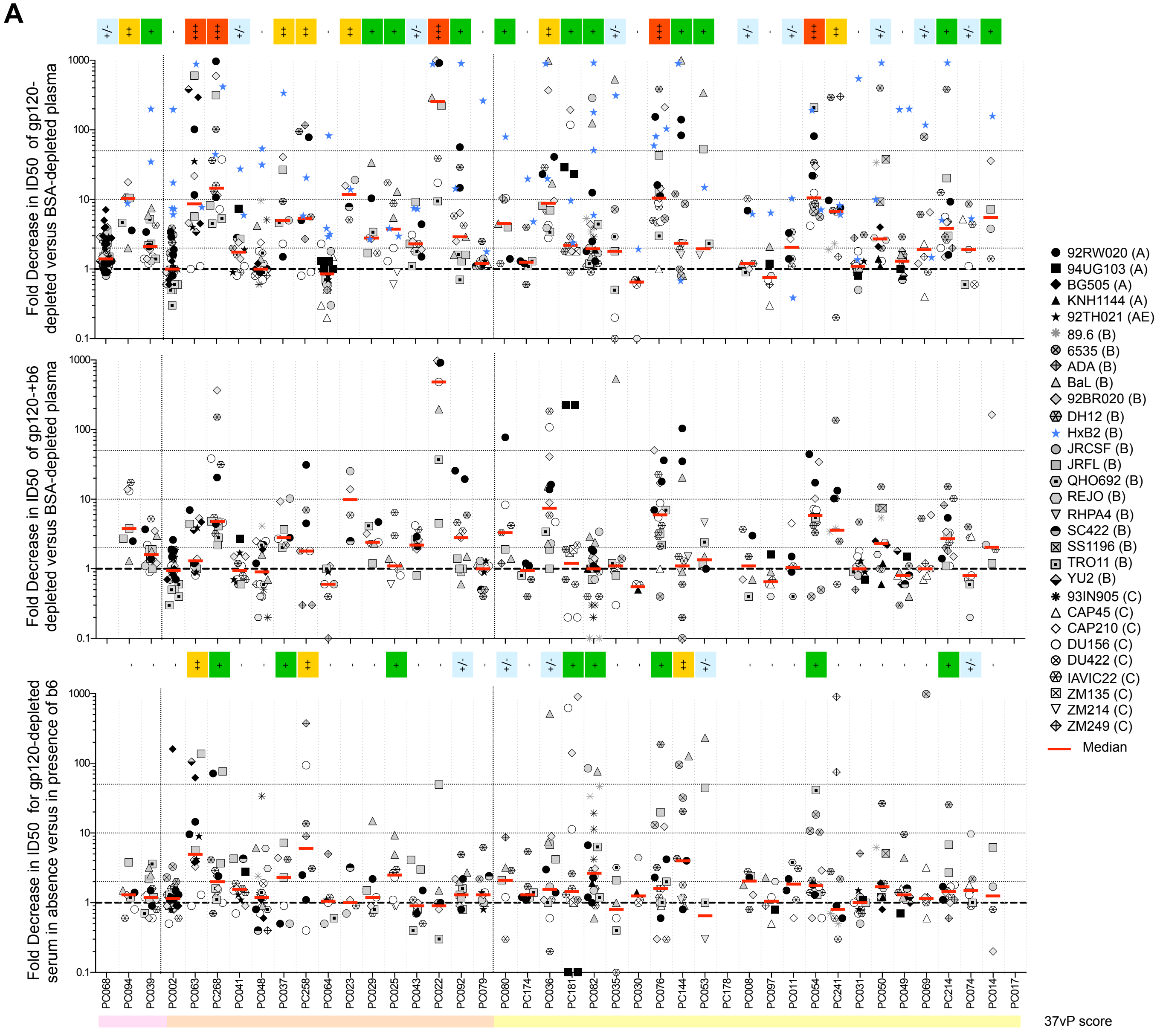

S8 Figure: Effect of recombinant gp120 absorption on the broad neutralizing activity of Protocol C neutralizers.

(A) Plasma from Top Protocol C Neutralizers were adsorbed with rgp120 coupled-beads in the presence (gp120+b6) or absence (gp120) of saturating concentration of b6 mAb or BSA control beads and then tested for neutralizing activity against a cross-clade pseudovirus panel (6 to 8 viruses). The strain of rgp120 used for depletion is indicated for each samples in S3 Table. The b6 competition was calculated as a fold decrease between the neutralization ID50 after rgp120 absorption in presence of b6 compared to the neutralization ID50 after rgp120 absorption in absence of b6. Participants are organized based on their neutralization score on the 37-virus panel. The overall effect of the depletion and strength of the b6 competition (b6 effect), as given is Fig 4A, is assigned based on (1) the median decrease in ID50 and (2) the fraction of viruses which neutralization ID50 was decreased >1.5 fold (+/-), >2 folds (+), >10 fold (++) or >50 fold (+++).

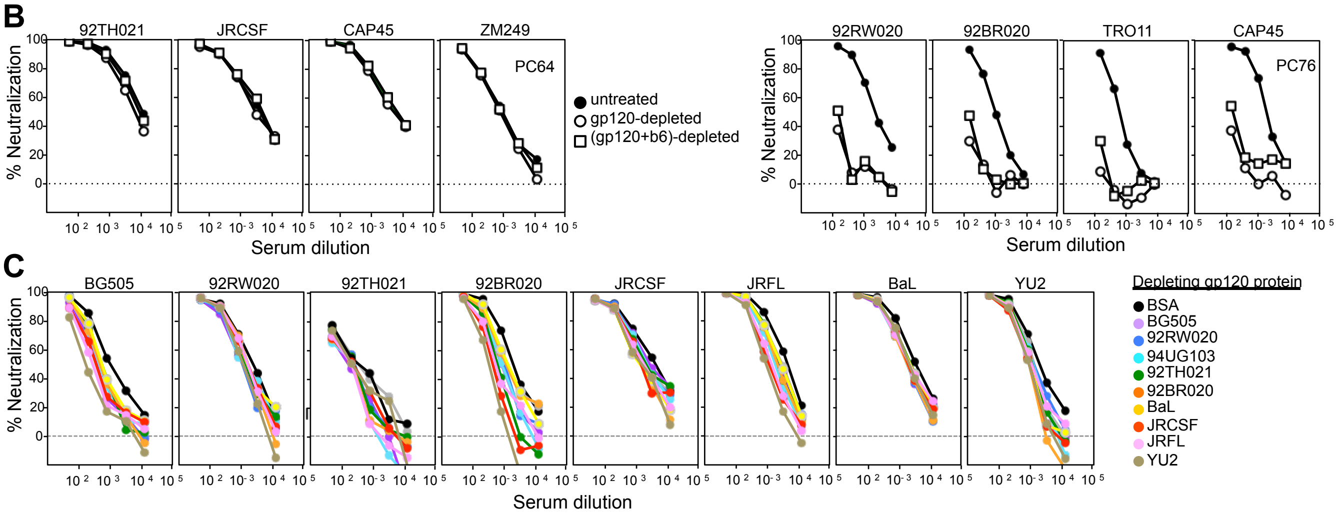

S8 Figure: Effect of recombinant gp120 absorption on the broad neutralizing activity of Protocol C neutralizers.
(B) Neutralization of HIV-1 pseudoviruses by titrated amount of plasma from participant PC064 (left) and PC076 (right) after adsorption on BSA- (black circles) or rgp120-coated beads in the absence of (open circles) or presence (open squares) of b6. (C) Neutralization of HIV-1 pseudoviruses by titrated amount of plasma from participant PC068 after adsorption on BSA-coated beads circles or beads coated with rgp120 from different strain of HIV-1.

A

Donor or mAb	HIV-2 WT ID50	HIV-2 C1 ID50	HIV-2 C3 ID50	HIV-2 C4 ID50	Category	Competition with MPER peptide on HIV-2 C1
PC068	NN	NN			-	
PC094	NN	246	147	NN	C3	78
PC039	176	221	78	112	WT	37
PC002	NN	219	NN	NN	C1	-5
PC063	NN	112	NN	195	C4	13
PC268	NN	NN			-	
PC041	NN	1798	NN	573	C4	23
PC048	NN	857	NN	297	C4	43
PC037	NN	600	NN	72	C4	39
PC258	NN	NN			-	
PC064	NN	1364	NN	237	C4	11
PC023	NN	NN			-	
PC029	NN	NN			-	
PC025	NN	NN			-	
PC043	NN	NN			-	
PC022	NN	NN			-	
PC092	NN	NN			-	
PC079	99	1131	127	671	C4	32
PC080	NN	NN			-	
PC174	NN	312	NN	257	C4	71
PC036	NN	NN			-	
PC181	NN	NN			-	
PC082	NN	538	NN	737	C4	-15
PC035	NN	2644	NN	664	C4	52
PC030	NN	NN			-	
PC076	NN	NN			-	
PC144	129	207	92	172	WT	-57
PC053	NN	NN			-	
PC178	64	328	239	212	C1	-88
PC008	NN	NN			-	
PC097	NN	968	NN	786	C4	72
PC011	NN	252	NN	NN	C1	86
PC054	NN	NN			-	
PC241	NN	NN	NN	NN	-	
PC031	NN	7471	NN	1600	C4	88
PC050	NN	161	NN	182	C4	-14
PC049	NN	NN			-	
PC069	NN	604	NN	NN	C1	33
PC214	NN	NN			-	
PC074	398	388	306	347	WT	12
PC014	NN	NN			-	
PC017	76	NN			-	
4E10	NN	0.061	NN	0.0865	C4	98
2F5	NN	0.0713	0.0526	NN	C3	100
Z13e1	NN	0.332	2.10	3.69		96

B

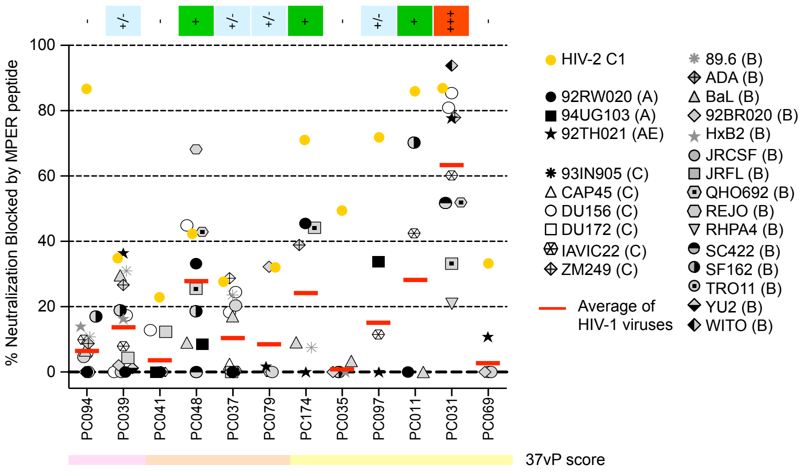

S9 Figure: Contribution of gp41 MPER-specific antibodies to broadly neutralizing antibody responses.
(A) Plasma from Protocol C top neutralizers were tested for neutralizing activity against chimeric HIV-2 pseudoviruses containing the complete (C1), partial N-terminal (C3: LALDKWASLW, 2F5 epitope) or C-terminal (C4: NWFDITKWLWYIK, 4E10 and 10E8 epitopes) MPER of HIV-1 YU2 gp41. The neutralization ID50 values are coded as follows: (blue) 50 ≤ ID50 <100; (yellow) 100 ≤ ID50 <300; (orange) 300≤ ID50 <900; (red) 900≤ ID50 <2700; (purple) ID50 ≥2700. Values below 50 are considered negative. NN indicates that the virus was not neutralized even at the lowest dilution of plasma tested (1:50). The last column records the percentage decrease of HIV-2 C1 neutralization in ID50 in the presence of 10mg/mL of MPER peptide, calculated using the equation: (1-($ID50_{presene\ of\ peptide}$- $ID50_{absence\ of\ peptide}$))*100 and color-coded as follows: (green) 25 to 50% decrease; (yellow) 50 to 75% decrease; (red) more than 75% decrease. Protocol C plasma samples are organized based on the neutralization on the 37-virus panel. (B) Plasma from Top Protocol C neutralizers were tested for neutralizing activity against HIV-2 C1 and several cross-clade HIV-1 pseudoviruses in the presence of 10mg/mL of MPER peptide. The percent decrease in neutralization ID50 in the presence of competitor is plotted and was calculated using the equation: (1-($ID50_{presene\ of\ peptide}$ $ID50_{absence\ of\ peptide}$))*100. Participants are listed and organized based on their neutralization score on the 37-virus panel. The overall strength of the MPER peptide competition, as given is Fig 4A, is assigned based on (1) the median decrease in ID50 and (2) the fraction of viruses which neutralization ID50 was decreased >20% (+/-), >40% (+), >60% (++), >80% fold (+++).

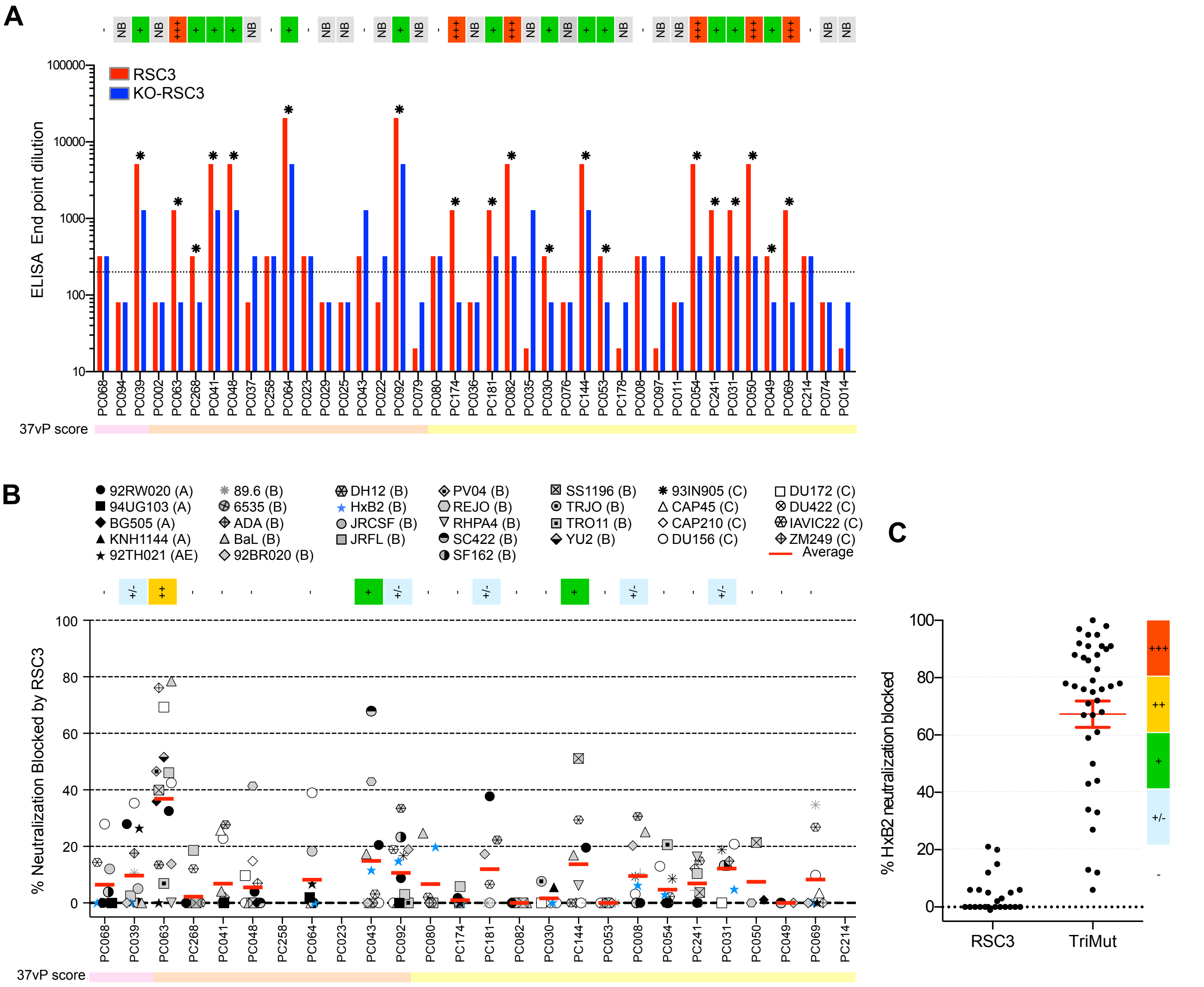

S10 Figure: Contribution of CD4 binding site-specific antibodies to broadly neutralizing antibody responses.
(A) Plasma from Protocol C top neutralizers were tested for binding to RSC3 and KO-RSC3 by ELISA. Protocol C plasma samples are organized based on their score on the 37v-panel. A star symbol is displayed when the End Point binding titer for RCS3 was at least 2.5 fold greater than for KO-RSC3. (B, C) Plasma from Top Protocol C Neutralizers were tested for neutralizing activity against cross-clade pseudoviruses in the presence of 25mg/mL RSC3/KO-RSC3 or TriMut/KO-TriMut. The percentage of neutralizing activity lost in the presence of competitor was calculated using the equation: $(1-(ID50_{core}-ID50_{Dcore}))*100$ and plotted. Protocol C plasma were competed by RSC3 for neutralization against several HIV-1 pseudoviruses (B) or on HxB2 only (C). nt: not tested. Participants are organized based on their neutralization score on the 37-virus panel. (D) Neutralization of HIV-1 pseudoviruses by titrated amount of plasma from participants PC053 and PC063, after adsorption on BSA- (black circles) or rgp120-coated beads, in the absence of (open circles) or presence (open squares) of b6. The overall strength of the RSC3 or TriMut competition, as given is Fig 4A, is assigned based on (1) the median decrease in ID50 and (2) the fraction of viruses which neutralization ID50 was decreased >20% (+/-), >40% (+), >60% (++), >80% fold (+++)

D

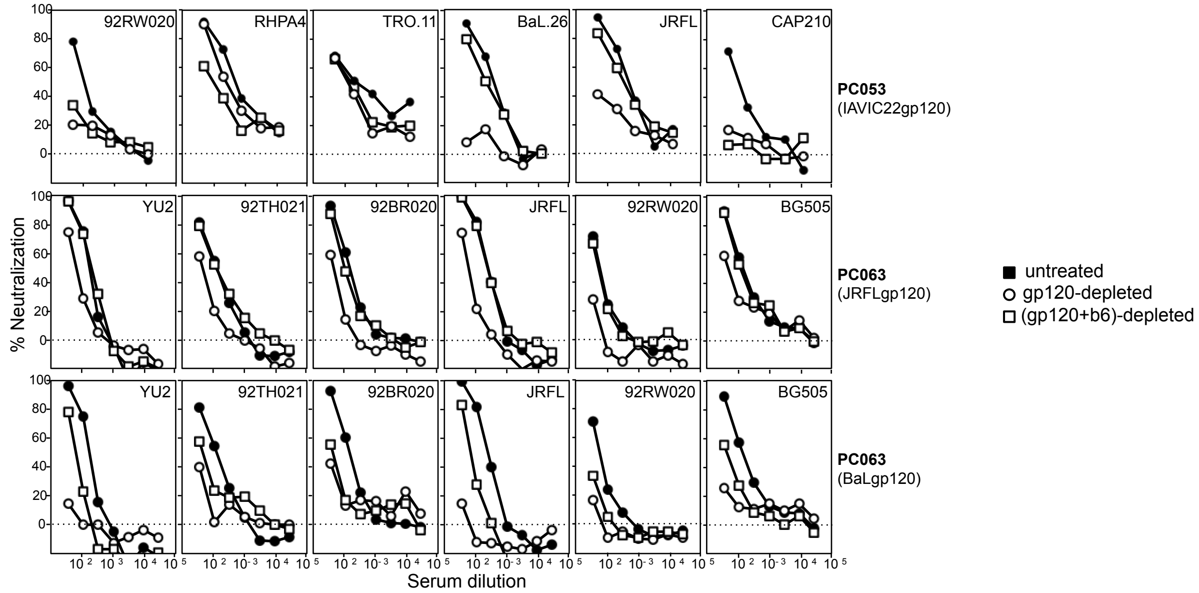

S10 Figure: Contribution of CD4 binding site-specific antibodies to broadly neutralizing antibody responses.
(D) Neutralization of HIV-1 pseudoviruses by titrated amount of plasma from participants PC053 and PC063, after adsorption on BSA- (black circles) or rgp120-coated beads, in the absence of (open circles) or presence (open squares) of b6.

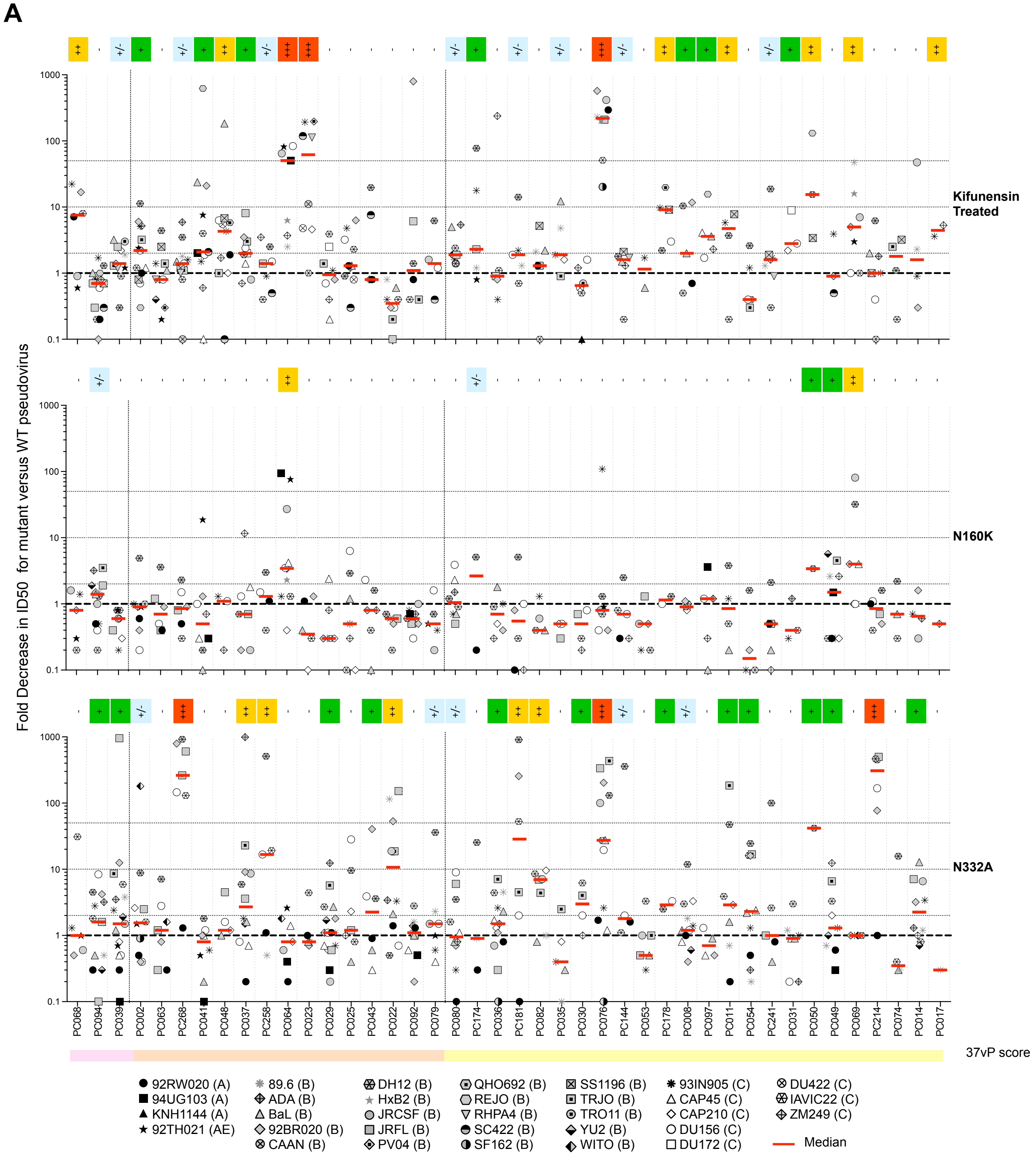

S11 Figure: Effect of kifunensin treatment and Env mutations on the broad neutralizing activity of Protocol C neutralizers.
(A) Plasma from top Protocol C neutralizers were tested for neutralizing activity against the indicated pseudoviruses produced in presence of kifunensin or carrying the indicated single or double mutations. The fold decrease in neutralization ID50 of mutant or treated virus compared to WT is plotted. The overall effect of the mutation and kifunensin treatment, as given is Fig 4A, is assigned based on (1) the median decrease in ID50 and (2) the fraction of viruses which neutralization ID50 was decreased >2 fold (+/-), >2 fold (+), >10 fold (++) or >50 fold (+++).

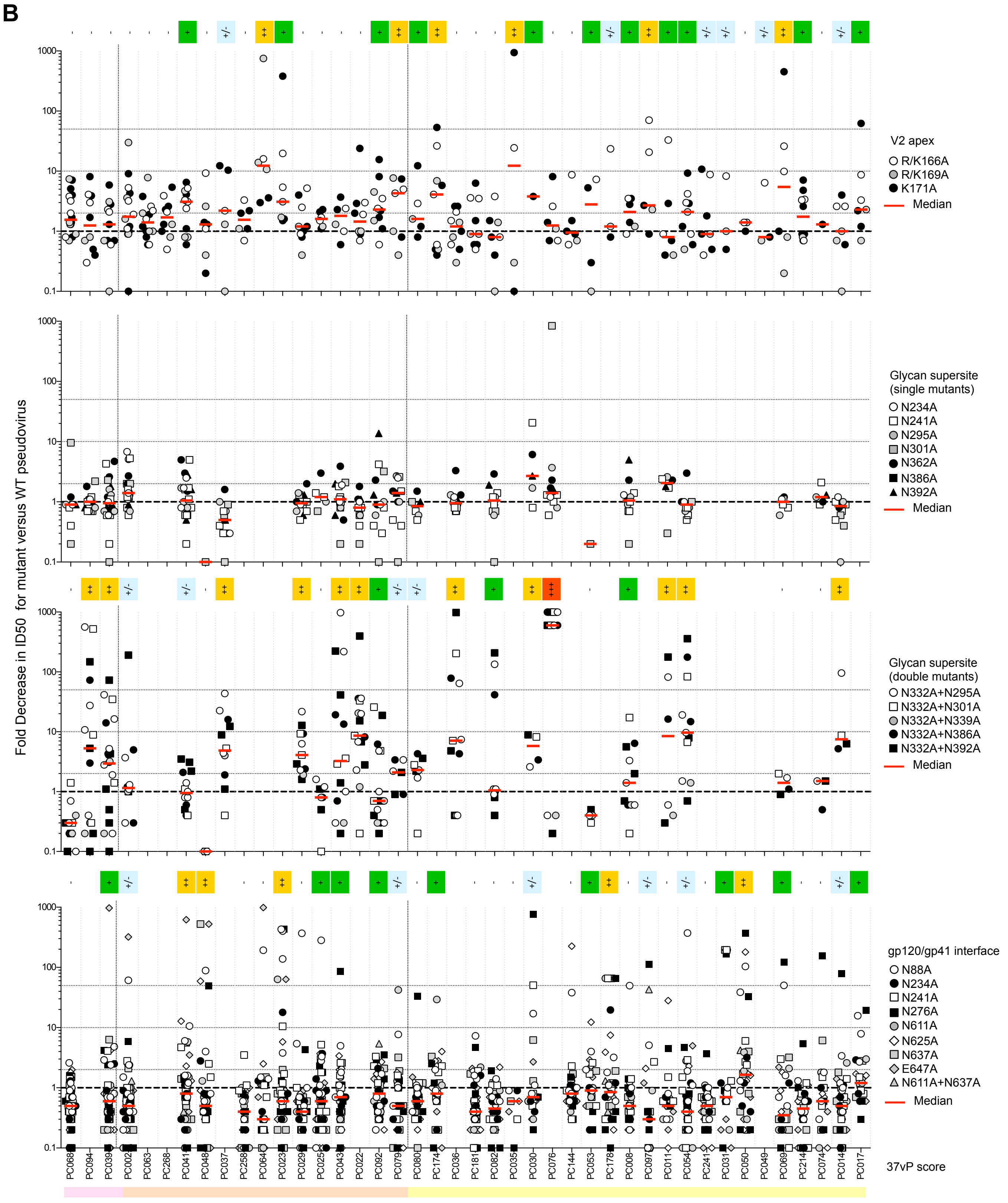

S11 Figure: Effect of kifunensin treatment and Env mutations on the broad neutralizing activity of Protocol C neutralizers.
(B) Plasma from top Protocol C neutralizers were tested for neutralizing activity against the indicated pseudoviruses carrying the indicated single or double mutations known to affect neutralization of monoclonal bnAbs targeting the V2 apex, glycan supersite (high-mannose patch) or gp120/gp41 interface epitopes. Each mutant was tested for 2-9 pseudoviruses when neutralized as WT (92RW020, 94UG103, BG505, 92TH021, 92BR020, JRCSF, JRFL, IAVIC22, 93IN905). The fold decrease in neutralization ID50 of mutant compared to WT is plotted. The overall effect of the mutations, as given is Fig 4A, is assigned based on (1) the median decrease in ID50 and (2) the fraction of viruses which neutralization ID50 was decreased >2 fold (+/-), >2 fold (+), >10 fold (++) or >50 fold (+++).

C

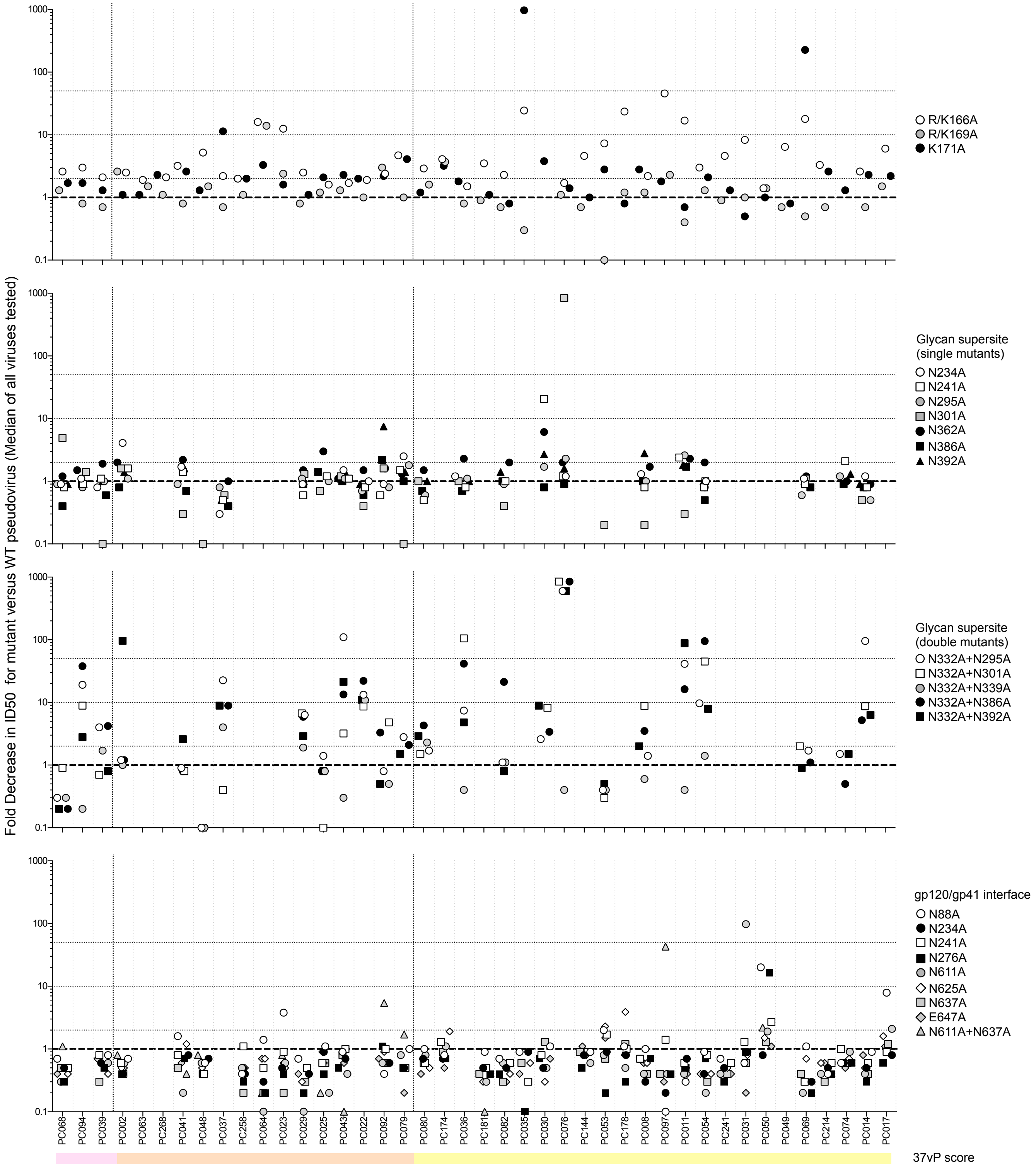

S11 Figure: Effect of kifunensin treatment and Env mutations on the broad neutralizing activity of Protocol C neutralizers.
(C) Plasma from top Protocol C neutralizers were tested for neutralizing activity against the indicated pseudoviruses carrying the indicated single or double mutations known to affect neutralization of monoclonal bnAbs targeting the V2 apex, glycan supersite (high-mannose patch) or gp120/gp41 interface epitopes. Each mutant was tested for 2-9 pseudoviruses when neutralized as WT (92RW020, 94UG103, BG505, 92TH021, 92BR020, JRCSF, JRFL, IAVIC22, 93IN905). The median fold decrease in neutralization ID50 of mutant compared to WT is plotted. Participants are organized based on their neutralization score on the 37-virus panel.

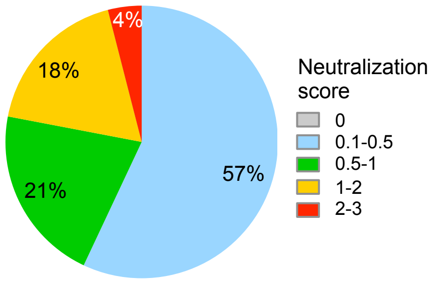

S12 Figure: Broadly neutralizing antibody responses in Protocol G. Plasma from sub-Saharan HIV-1 infected participants (N>1500) enrolled in the cross-sectional IAVI Protocol G study and collected between 3-10 years post infection were assessed for neutralizing activity on a predictive 6v-panel [16]. Neutralization score on the 6v-panel was calculated as indicated in Material and Methods.

S1 Table: Protocol C cohort demography

* MSM: Men who report sex with men. ** Recombinant subtypes in the pol region include A1/A2/D (2), A1/C (6), A1/C/D (1), A1/D (14), C/K (1), CRF02_AG (2), CRF11_CPX (1), and D/C (1).

	Whole cohort		Studied subset		p value ∞	Studied subset		M48+ subset		p value ∞
	N	%	N	%		N	%	N	%	
Total	613	100	439	100		439	100	228	100	
Volunteer sex					0.99					0.99
Female	255	41.6	178	40.5		178	40.5	93	40.8	
Male	358	58.4	261	59.5		261	59.5	135	59.2	
Transmission route					0.99					0.99
Discordant couples	442	72.1	323	73.6		323	73.6	169	74.1	
Other heterosexual risk	71	11.6	47	10.7		47	10.7	23	10.1	
MSM*	92	15.0	62	14.1		62	14.1	30	13.2	
Don't know/unknown	8	1.3	7	1.6		7	1.6	6	2.6	
Age at time of EDI					0.60					0.40
Mean, median (SD)	30.6, 29	(8.5)	30.3, 29	(8.3)		30.3, 29	(8.3)	31.5, 30	(8.7)	
Body Mass Index at enrollment					0.07					0.39
<18.5	103	16.8	65	14.8		65	14.8	30	13.2	
18.5-25	432	70.5	313	71.3		313	71.3	169	74.1	
>25	78	12.7	61	13.9		61	13.9	29	12.7	
Clinical Research Center					0.99					0.97
Kilifi (Kenya)	88	14.4	55	12.5		55	12.5	30	13.2	
Kangemi (Kenya)	25	4.1	21	4.8		21	4.8	8	3.5	
Kigali (Rwanda)	94	15.3	71	16.2		71	16.2	43	18.9	
Cape Town (South Africa)	7	1.1	3	0.7		3	0.7	0	0.0	
Rustenburg (South Africa)	22	3.6	11	2.5		11	2.5	0	0.0	
Masaka (Uganda)	97	15.8	82	18.7		82	18.7	49	21.5	
Entebbe (Uganda)	46	7.5	24	5.5		24	5.5	11	4.8	
Lusaka (Zambia)	151	24.6	117	26.7		117	26.7	62	27.2	
Copperbelt (Zambia)	83	13.5	54	12.3		54	12.3	25	11.0	
Country					0.97					0.96
Kenya	113	18.4	76	17.3		76	17.3	38	16.7	
Rwanda	94	15.3	71	16.2		71	16.2	43	18.9	
South Africa	29	4.7	14	3.2		14	3.2	0	0.0	
Uganda	143	23.3	106	24.1		106	24.1	60	26.3	
Zambia	234	38.2	171	39.0		171	39.0	87	38.2	
Set point viral load (log10 copies/mL)					0.3					0.02
Mean, median (SD)	4.48, 4.59	(0.92)	4.42, 4.53	(0.92)		4.42, 4.53	(0.92)	4.25, 4.34	(0.95)	
Missing	16	2.6	3	0.7		3	0.7	3	1.3	
Set point CD4 T cell count (cells/μL)					0.02					0.02
Mean, median (SD)	579.6, 534	(243.5)	610.3,579	(238.5)		610.3,579	(238.5)	652.2, 631	(247.4)	
Missing	15	2.4	4	0.9		4	0.9	2	0.9	
Infecting HIV-1 subtype (*pol*)					0.73					0.96
A1	214	34.9	153	34.9		153	34.9	93	40.8	
B	1	0.2	1	0.2		1	0.2	1	0.4	
C	277	45.2	202	46.0		202	46.0	94	41.2	
D	85	13.9	62	14.1		62	14.1	32	14.0	
G	2	0.3	1	0.2		1	0.2	0	0.0	
Recombinant**	29	4.7	17	3.9		17	3.9	7	3.1	
Missing	5	0.8	1	0.2		1	0.2	1	0.4	
Study status										
Still on study	155	25.3	168	38.3	0.72	168	38.3	116	50.9	0.70
Off study (reasons below:)	458	74.7	271	61.7		271	61.7	112	49.1	
ART	277	45.2	202	46.0		202	46.0	102	44.7	
Unknown	64	10.4	32	7.3		32	7.3	8	3.5	
Moved	29	4.7	9	2.1		9	2.1	5	2.2	
Death, not AIDS related	15	2.4	11	2.5		11	2.5	6	2.6	
Investigator discretion	10	1.6	4	0.9		4	0.9	1	0.4	
Volunteer discretion	17	2.8	5	1.1		5	1.1	4	1.8	
Other	5	0.8	4	0.9		4	0.9	4	1.8	
Death, AIDS related	8	1.3	6	1.4		6	1.4	2	0.9	
Site closure/study end	33	5.4	21	4.8		21	4.8	5	2.2	
ART-free person years on study, mean, median, (SD)	3.87, 3.73	(2.11)	4.76, 4.67	(1.73)	<0.001	4.76, 4.67	(1.73)	5.94, 5.96	(1.32)	<0.001

∞ p value comparing those in the subset to those not in the subset

Donor	Neutralization score at each visit														Best	Last TP	37v	37v Panel
	18	21	24	30	36	42	48	54	60	66	72	78	84	90				
PC068	ND	ND	0.17	0.33	0.33	1.00	1.33	2.17	2.33	2.33	ND	ND	ND	ND	2.33	66	3.31	***60***
PC157	ND	ND	0.00	0.33	0.33	0.67	0.67	0.83	1.33	1.33	2.17	1.67	ND	ND	2.17	78		
PC092	ND	ND	0.17	0.67	1.00	1.50	1.50	1.83	1.67	2.17	ND	ND	ND	ND	2.17	66	1.51	***36***
PC039	ND	ND	1.67	1.33	1.67	1.50	1.67	1.67	2.00	ND	ND	ND	ND	ND	2.00	60	2.38	***36***
PC064	0.50	1.50	1.33	1.83	2.00	1.50	1.33	ND	ND	ND	ND	ND	ND	ND	2.00	48	1.72	***36***
PC386	ND	ND	1.50	1.17	1.50	2.00	ND	ND	ND	ND	ND	ND	ND	ND	2.00	42		
PC329	ND	ND	0.83	0.83	2.00	ND	ND	ND	ND	ND	ND	ND	ND	ND	2.00	36		
PC023	ND	ND	0.00	0.17	0.17	0.17	0.17	0.00	0.67	1.50	1.67	1.83	ND	1.50	1.83	90	0.98	***60***
PC054	ND	ND	0.50	1.50	1.00	1.17	1.33	1.33	ND	1.33	1.83	0.00	ND	ND	1.83	78	1.25	***36***
PC053	ND	ND	0.00	0.00	0.17	0.17	0.50	1.00	1.83	1.00	ND	ND	ND	ND	1.83	66	1.31	***48***
PC094	ND	ND	ND	0.50	1.00	1.33	1.67	1.33	0.83	1.67	ND	1.67	ND	ND	1.67	78	2.17	***48***
PC268	ND	ND	0.17	0.17	1.33	1.67	ND	ND	ND	ND	ND	ND	ND	ND	1.67	42	1.87	***36***
PC035	ND	ND	0.17	0.33	1.50	0.50	0.83	1.00	1.17	ND	ND	ND	ND	ND	1.50	60	1.41	***36***
PC022	ND	ND	0.00	ND	0.33	1.33	1.50	ND	ND	ND	ND	ND	ND	ND	1.50	48	1.52	***48***
PC008	ND	ND	0.33	0.33	0.33	1.00	0.67	0.83	1.33	1.33	0.83	0.83	1.00	ND	1.33	84	1.28	***54***
PC174	ND	ND	0.00	0.33	0.67	ND	1.33	0.00	ND	ND	ND	ND	ND	ND	1.33	54	1.44	***48***
PC036	0.67	0.83	0.67	1.17	0.83	1.17	1.33	ND	ND	ND	ND	ND	ND	ND	1.33	48	1.43	***30***
PC214	ND	ND	1.33	1.17	1.00	0.83	ND	ND	ND	ND	ND	ND	ND	ND	1.33	42	1.05	***30***
PC397	ND	ND	1.00	1.33	0.00	ND	ND	ND	ND	ND	ND	ND	ND	ND	1.33	36		
PC041	ND	ND	0.00	0.00	0.33	0.33	0.83	0.17	0.83	1.17	1.17	0.83	ND	ND	1.17	78	1.86	***36***
PC030	ND	ND	0.00	0.00	0.67	0.50	0.67	0.83	1.17	ND	0.33	0.83	ND	ND	1.17	78	0.91	***48***
PC076	ND	ND	0.17	1.00	1.00	1.17	1.00	1.00	0.67	0.67	ND	ND	ND	ND	1.17	66	1.32	***36***
PC025	ND	ND	0.33	1.00	0.83	0.67	0.17	1.17	0.67	ND	ND	ND	ND	ND	1.17	60	1.53	***36***
PC048	ND	ND	ND	0.50	0.33	0.50	0.83	1.17	ND	ND	ND	ND	ND	ND	1.17	54	1.26	***48***
PC181	ND	ND	0.50	0.83	1.00	1.00	1.00	1.17	ND	ND	ND	ND	ND	ND	1.17	54	1.43	***48***
PC082	ND	ND	0.67	0.67	0.50	1.17	0.50	ND	ND	ND	ND	ND	ND	ND	1.17	48	1.41	***30***
PC002	ND	ND	0.67	1.17	1.00	1.00	1.17	ND	ND	ND	ND	ND	ND	ND	1.17	48	1.99	***36***
PC029	ND	ND	0.00	0.00	0.00	0.00	1.17	ND	ND	ND	ND	ND	ND	ND	1.17	48	1.67	***48***
PC037	ND	ND	0.33	0.67	1.17	0.83	ART	ND	ND	ND	ND	ND	ND	ND	1.17	42	1.80	***36***
PC387	ND	ND	1.17	ND	1.17	1.17	ND	ND	ND	ND	ND	ND	ND	ND	1.17	42		
PC258	ND	ND	0.67	1.17	1.17	ART	ND	ND	ND	ND	ND	ND	ND	ND	1.17	36	1.78	***30***
PC303	ND	ND	0.50	0.50	1.17	ND	ND	ND	ND	ND	ND	ND	ND	ND	1.17	36		
PC439	ND	ND	1.17	0.83	ND	ND	ND	ND	ND	ND	ND	ND	ND	ND	1.17	30		
PC063	ND	ND	0.00	0.00	0.00	0.00	0.00	0.00	0.00	0.67	0.83	1.00	ND	ND	1.00	78	1.89	***72***
PC067	ND	ND	0.17	0.50	0.17	0.33	0.33	0.50	0.50	0.50	1.00	ND	ND	ND	1.00	72	0.64	***30***
PC011	ND	ND	0.17	0.33	0.50	0.83	0.67	0.50	0.67	1.00	1.00	ND	ND	ND	1.00	72	1.26	***42***
PC014	ND	ND	0.00	0.33	0.67	0.67	1.00	0.83	0.67	0.83	1.00	ND	ND	ND	1.00	72	1.03	***48***
PC130	ND	ND	0.00	0.50	0.33	0.83	0.33	0.50	0.67	0.83	1.00	ND	ND	ND	1.00	72		
PC017	0.83	1.00	1.00	0.67	0.33	ND	0.67	0.50	0.50	1.00	ND	ND	ND	ND	1.00	66	0.62	***36***
PC080	ND	ND	0.50	1.00	0.83	1.00	0.83	1.00	0.83	1.00	ND	ND	ND	ND	1.00	66	1.48	***48***
PC104	ND	ND	0.00	0.50	0.67	0.50	0.83	0.83	1.00	0.83	ND	ND	ND	ND	1.00	66	0.95	***60***
PC196	ND	ND	0.00	0.00	0.33	0.33	1.00	1.00	ND	ND	ND	ND	ND	ND	1.00	54		
PC264	ND	ND	0.00	0.17	0.17	0.67	1.00	0.50	ND	ND	ND	ND	ND	ND	1.00	54		
PC241	ND	ND	0.50	1.00	0.83	1.00	0.50	ND	ND	ND	ND	ND	ND	ND	1.00	48	1.25	***36***
PC192	ND	ND	0.50	0.67	1.00	0.50	ND	ND	ND	ND	ND	ND	ND	ND	1.00	42	0.96	***36***
PC311	ND	ND	0.67	0.67	1.00	1.00	ND	ND	ND	ND	ND	ND	ND	ND	1.00	42		
PC003	ND	ND	0.17	0.00	0.17	0.00	0.00	0.33	0.17	ND	0.33	0.50	0.83	ND	0.83	84	0.48	***54***
PC129	ND	ND	0.17	0.33	0.33	0.00	0.17	0.33	0.17	ND	0.33	0.17	0.83	ND	0.83	84		
PC113	ND	ND	0.00	0.00	ND	0.17	0.17	0.17	0.00	0.17	0.83	0.00	ND	ND	0.83	78		
PC159	ND	ND	0.33	0.33	0.17	0.33	0.33	0.33	0.83	0.17	0.33	0.83	ND	ND	0.83	78		
PC031	ND	ND	0.17	0.83	0.67	0.83	0.83	0.67	0.83	0.83	0.83	ND	ND	ND	0.83	72	1.17	***36***
PC115	ND	ND	0.00	0.50	0.17	ND	0.33	0.67	0.50	0.83	0.67	ND	ND	ND	0.83	72		
PC175	ND	0.00	ND	0.33	0.33	0.17	0.33	ND	0.33	ND	0.83	ND	ND	ND	0.83	72		
PC213	ND	ND	ND	0.00	0.17	0.00	ND	0.00	0.00	0.50	0.83	ND	ND	ND	0.83	72		
PC024	ND	ND	0.00	0.33	0.50	0.67	0.50	0.50	0.67	0.83	ND	ND	ND	ND	0.83	66	0.67	***48***
PC050	ND	ND	0.33	0.00	0.33	0.00	0.50	0.83	0.67	ND	ND	ND	ND	ND	0.83	60	1.17	***54***
PC140	ND	ND	0.17	0.17	0.50	0.67	0.67	0.83	ND	ND	ND	ND	ND	ND	0.83	54		
PC149	ND	ND	0.33	0.83	0.67	0.33	0.33	0.33	ART	ND	ND	ND	ND	ND	0.83	54		
PC151	ND	ND	0.00	0.00	0.17	0.33	0.67	0.83	ND	ND	ND	ND	ND	ND	0.83	54		
PC043	ND	ND	0.17	0.83	0.83	0.67	0.50	ND	ND	ND	ND	ND	ND	ND	0.83	48	1.57	***36***
PC016	ND	ND	ND	0.33	0.83	0.50	0.50	ND	ND	ND	ND	ND	ND	ND	0.83	48	0.71	***36***
PC178	ND	ND	ND	0.17	0.50	0.83	0.33	ND	ND	ND	ND	ND	ND	ND	0.83	48	1.30	***42***
PC309	ND	ND	0.33	0.83	ND	0.83	0.67	ND	ND	ND	ND	ND	ND	ND	0.83	48		
PC315	ND	0.17	ND	0.00	0.17	0.33	0.83	ND	ND	ND	ND	ND	ND	ND	0.83	48		
PC177	ND	ND	ND	0.33	0.83	0.50	0.33	ND	ND	ND	ND	ND	ND	ND	0.83	48		
PC044	0.33	0.33	0.83	0.33	0.83	ART	ND	ND	ND	ND	ND	ND	ND	ND	0.83	36	0.89	***24***
PC077	ND	ND	0.00	0.33	0.00	0.17	0.00	0.00	0.00	0.17	0.17	0.67	0.33	0.67	0.67	90	0.80	***78***
PC010	ND	ND	0.17	0.50	0.67	0.50	0.67	0.50	0.17	0.33	0.33	0.50	0.67	0.00	0.67	90		
PC012	ND	ND	0.50	0.33	0.33	0.00	0.33	0.33	ND	0.67	0.17	0.67	ND	ND	0.67	78	0.99	***48***
PC152	ND	ND	0.33	0.50	0.50	0.33	0.33	0.33	0.00	0.17	0.67	0.17	ND	ND	0.67	78		
PC042	ND	ND	0.00	0.50	0.17	0.50	0.67	0.33	0.33	0.33	0.00	ND	ND	ND	0.67	72	0.76	***48***
PC062	ND	ND	0.17	0.17	0.33	0.33	0.50	0.67	0.33	ND	0.50	ND	ND	ND	0.67	72		
PC065	ND	ND	0.33	0.33	0.00	0.33	0.67	0.50	0.00	0.33	0.33	ND	ND	ND	0.67	72		
PC097	ND	ND	0.50	0.17	0.33	0.67	0.67	0.33	ND	0.33	ND	ND	ND	ND	0.67	66	1.28	***42***
PC254	ND	ND	0.00	0.33	0.33	ND	0.33	0.50	0.67	0.67	ND	ND	ND	ND	0.67	66		
PC109	ND	ND	0.00	0.00	0.33	0.00	ND	0.67	0.17	0.33	ND	ND	ND	ND	0.67	66		
PC132	ND	ND	0.50	0.33	0.17	0.33	0.33	0.33	0.67	0.33	ND	ND	ND	ND	0.67	66		
PC049	ND	ND	0.00	ND	0.17	0.33	ND	0.67	0.33	ART	ND	ND	ND	ND	0.67	60	0.96	***36***
PC032	0.17	ND	0.17	0.33	0.67	0.33	0.50	0.17	0.33	ND	ND	ND	ND	ND	0.67	60	0.74	***36***
PC163	ND	ND	0.00	0.17	0.33	0.50	0.67	0.33	0.50	ND	ND	ND	ND	ND	0.67	60		
PC314	ND	ND	ND	0.00	0.67	ND	ND	0.17	0.33	ND	ND	ND	ND	ND	0.67	60		
PC128	ND	ND	0.00	0.67	0.33	0.33	0.33	0.33	0.33	ND	ND	ND	ND	ND	0.67	60		
PC045	0.00	0.17	0.17	0.33	0.33	0.00	0.33	0.67	ART	ART	ART	ART	ART	ND	0.67	54	0.29	***30***
PC144	ND	ND	0.00	ND	ND	0.67	0.50	0.67	ND	ND	ND	ND	ND	ND	0.67	54	1.31	***54***
PC338	ND	ND	0.17	0.33	0.50	0.17	0.67	0.33	ND	ND	ND	ND	ND	ND	0.67	54		
PC001	ND	ND	0.00	0.67	0.50	0.50	0.67	ND	ND	ND	ND	ND	ND	ND	0.67	48	0.98	***36***
PC195	ND	ND	0.00	0.17	0.00	0.67	0.67	ART	ND	ND	ND	ND	ND	ND	0.67	48		
PC238	ND	ND	0.00	0.33	0.17	0.17	0.67	ND	ND	ND	ND	ND	ND	ND	0.67	48		
PC251	ND	ND	0.00	0.33	0.33	0.50	0.67	ND	ND	ND	ND	ND	ND	ND	0.67	48		
PC230	ND	ND	0.00	0.17	0.17	0.33	0.67	ND	ND	ND	ND	ND	ND	ND	0.67	48		
PC233	ND	ND	ND	ND	0.67	0.33	ART	ND	ND	ND	ND	ND	ND	ND	0.67	42	1.00	***36***
PC388	ND	ND	ND	0.00	0.33	0.67	ND	ND	ND	ND	ND	ND	ND	ND	0.67	42		
PC401	ND	ND	0.00	0.17	ND	0.67	ND	ND	ND	ND	ND	ND	ND	ND	0.67	42		
PC255	ND	ND	0.00	0.67	0.33	ND	ND	ND	ND	ND	ND	ND	ND	ND	0.67	36		
PC256	ND	ND	0.00	0.17	0.67	ND	ND	ND	ND	ND	ND	ND	ND	ND	0.67	36		
PC368	ND	ND	0.00	0.17	0.67	ND	ND	ND	ND	ND	ND	ND	ND	ND	0.67	36		
PC280	ND	ND	ND	ND	0.67	ND	ND	ND	ND	ND	ND	ND	ND	ND	0.67	36		
PC390	ND	ND	0.33	0.67	0.50	ND	ND	ND	ND	ND	ND	ND	ND	ND	0.67	36		
PC079	ND	ND	0.50	0.67	ND	ND	ND	ND	ND	ND	ND	ND	ND	ND	0.67	30	1.51	***30***
PC322	ND	ND	0.67	0.67	ND	ND	ND	ND	ND	ND	ND	ND	ND	ND	0.67	30		
PC454	ND	ND	0.67	ND	ND	ND	ND	ND	ND	ND	ND	ND	ND	ND	0.67	24		
PC034	ND	ND	0.00	0.33	0.33	0.50	0.17	0.17	0.17	0.33	0.33	0.33	0.50	ND	0.50	84		
PC018	ND	ND	ND	0.00	0.33	0.00	0.00	0.17	0.33	0.00	0.17	0.17	0.50	ND	0.50	84		
PC028	ND	ND	0.00	0.33	0.33	0.00	0.17	0.50	0.17	ND	0.33	0.33	0.00	ND	0.50	84		
PC071	ND	ND	0.17	0.33	0.33	0.50	0.17	0.17	0.17	ND	0.33	0.50	0.50	ND	0.50	84		
PC072	ND	ND	0.50	0.50	0.50	0.00	0.33	0.33	0.33	0.33	0.50	0.33	0.33	ND	0.50	84		
PC015	ND	ND	0.33	0.50	0.33	ND	0.17	0.00	0.50	ND	ND	0.50	ND	ND	0.50	78	0.63	***48***
PC114	ND	0.17	ND	0.17	0.33	0.17	0.50	0.33	0.50	ND	ND	0.17	ND	ND	0.50	78		
PC087	ND	ND	0.17	0.33	0.50	0.17	0.50	0.17	0.33	0.00	0.00	0.17	ND	ND	0.50	78		
PC089	ND	ND	0.00	0.33	0.33	0.17	0.17	0.50	0.33	0.33	0.50	0.50	ND	ND	0.50	78		
PC205	ND	ND	ND	0.00	0.17	0.17	ND	ND	0.50	ND	0.33	0.33	ND	ND	0.50	78		
PC108	ND	ND	0.00	0.17	0.00	0.17	0.00	0.00	0.17	0.00	0.00	0.50	ND	ND	0.50	78		
PC075	ND	ND	0.00	0.00	0.33	0.00	0.50	0.33	0.50	ND	0.33	ND	ND	ND	0.50	72	0.68	***36***
PC116	ND	ND	0.00	0.00	0.00	0.17	0.00	0.00	0.17	0.50	0.00	ND	ND	ND	0.50	72		
PC083	ND	ND	0.00	0.17	0.00	0.50	ND	ND	0.17	0.00	0.00	ND	ND	ND	0.50	72		
PC069	ND	ND	0.00	0.00	0.33	0.33	0.33	0.50	0.00	0.17	ND	ND	ND	ND	0.50	66	1.08	***48***
PC061	ND	ND	0.17	0.17	0.00	0.50	0.17	0.17	0.00	0.00	ND	ND	ND	ND	0.50	66	0.59	***54***
PC091	ND	ND	0.00	0.00	0.17	0.00	0.17	0.50	0.33	0.17	ND	ND	ND	ND	0.50	66		
PC142	ND	ND	0.17	0.17	0.33	0.50	0.00	0.17	0.00	0.00	ND	ND	ND	ND	0.50	66		
PC146	ND	ND	0.00	0.00	ND	0.00	0.00	0.00	0.50	0.33	ND	ND	ND	ND	0.50	66		
PC184	ND	ND	0.00	0.00	0.33	0.00	0.33	0.33	0.17	0.50	ND	ND	ND	ND	0.50	66		
PC110	ND	ND	0.33	0.17	0.33	0.33	0.50	0.33	0.33	0.33	ND	ND	ND	ND	0.50	66		
PC133	ND	ND	0.00	0.17	0.50	0.33	0.33	0.17	0.50	0.00	ND	ND	ND	ND	0.50	66		
PC119	ND	ND	0.17	0.00	0.00	0.50	0.17	0.17	0.17	ND	ND	ND	ND	ND	0.50	60		
PC126	ND	ND	0.00	0.33	0.33	0.33	0.50	0.50	0.50	ND	ND	ND	ND	ND	0.50	60		
PC127	ND	ND	0.00	0.00	0.00	0.33	0.50	0.33	0.33	ND	ND	ND	ND	ND	0.50	60		
PC197	ND	ND	0.17	0.17	0.17	0.33	0.50	0.33	ND	ND	ND	ND	ND	ND	0.50	54		
PC199	ND	ND	0.33	0.17	0.33	0.17	0.50	0.33	ND	ND	ND	ND	ND	ND	0.50	54		
PC210	ND	ND	0.17	0.17	0.17	0.50	0.50	0.33	ND	ND	ND	ND	ND	ND	0.50	54		
PC246	ND	ND	ND	0.33	0.17	0.50	0.33	0.33	ND	ND	ND	ND	ND	ND	0.50	54		
PC226	ND	ND	0.00	0.33	0.17	0.50	0.50	0.00	ND	ND	ND	ND	ND	ND	0.50	54		
PC158	ND	ND	0.17	0.17	0.17	0.50	0.33	0.33	ND	ND	ND	ND	ND	ND	0.50	54		
PC231	ND	ND	0.17	0.17	0.17	0.17	0.50	0.17	ND	ND	ND	ND	ND	ND	0.50	54		
PC074	ND	0.00	ND	ND	0.17	0.00	0.50	ND	ND	ND	ND	ND	ND	ND	0.50	48	1.04	***48***
PC267	ND	ND	0.17	0.17	0.17	0.50	0.33	ND	ND	ND	ND	ND	ND	ND	0.50	48		
PC270	ND	ND	0.17	0.17	0.00	0.17	0.50	ND	ND	ND	ND	ND	ND	ND	0.50	48		
PC284	ND	ND	0.00	0.00	0.00	0.00	0.50	ART	ND	ND	ND	ND	ND	ND	0.50	48		
PC026	ND	ND	0.00	0.00	0.50	0.33	ND	ND	ND	ND	ND	ND	ND	ND	0.50	42	0.39	***36***
PC189	ND	ND	0.33	0.17	0.50	0.00	ART	ND	ND	ND	ND	ND	ND	ND	0.50	42		
PC206	ND	ND	ND	0.50	0.17	0.00	ND	ND	ND	ND	ND	ND	ND	ND	0.50	42		
PC209	ND	ND	0.00	0.33	0.50	0.17	ART	ND	ND	ND	ND	ND	ND	ND	0.50	42		
PC223	ND	ND	0.33	0.33	0.17	0.50	ND	ND	ND	ND	ND	ND	ND	ND	0.50	42		
PC389	ND	ND	0.33	0.50	0.50	0.00	ND	ND	ND	ND	ND	ND	ND	ND	0.50	42		
PC365	ND	ND	0.50	0.17	ND	0.33	ND	ND	ND	ND	ND	ND	ND	ND	0.50	42		
PC227	ND	ND	0.00	0.50	0.00	0.00	ND	ND	ND	ND	ND	ND	ND	ND	0.50	42		
PC229	ND	ND	0.33	0.50	0.50	0.33	ND	ND	ND	ND	ND	ND	ND	ND	0.50	42		
PC234	ND	ND	0.50	0.50	0.33	ND	ND	ND	ND	ND	ND	ND	ND	ND	0.50	36		
PC236	ND	ND	0.50	0.00	0.50	ND	ART	ART	ND	ND	ND	ND	ND	ND	0.50	36		
PC239	ND	ND	0.33	0.50	0.17	ND	ND	ND	ND	ND	ND	ND	ND	ND	0.50	36		
PC253	ND	ND	ND	0.33	0.50	ND	ND	ND	ND	ND	ND	ND	ND	ND	0.50	36		
PC259	ND	ND	0.50	0.33	0.17	ND	ND	ND	ND	ND	ND	ND	ND	ND	0.50	36		
PC375	ND	ND	0.33	0.50	0.33	ND	ND	ND	ND	ND	ND	ND	ND	ND	0.50	36		
PC312	ND	ND	0.50	0.50	ART	ART	ND	ND	ND	ND	ND	ND	ND	ND	0.50	30		
PC316	ND	ND	0.50	0.00	ND	ND	ND	ND	ND	ND	ND	ND	ND	ND	0.50	30		
PC337	ND	ND	0.17	0.50	ART	ART	ND	ND	ND	ND	ND	ND	ND	ND	0.50	30		
PC385	ND	ND	0.00	0.50	ND	ND	ND	ND	ND	ND	ND	ND	ND	ND	0.50	30		
PC453	ND	ND	0.50	ART	ND	ND	ND	ND	ND	ND	ND	ND	ND	ND	0.50	24		
PC051	0.00	0.00	0.00	0.33	0.33	0.17	0.17	0.00	ND	ND	ND	ND	ND	ND	0.33	54	0.39	***30***
PC081	ND	ND	0.17	0.17	0.33	0.17	0.33	0.33	ND	ND	ND	ND	ND	ND	0.33	54	0.98	***42***
PC060	ND	ND	0.17	0.17	0.00	0.17	0.33	0.33	ND	ND	ND	ND	ND	ND	0.33	54	0.88	***54***
PC033	ND	ND	0.17	0.33	0.33	0.17	ND	ND	ND	ND	ND	ND	ND	ND	0.33	42	0.90	***36***
PC019	0.33	0.33	0.33	0.17	ART	ART	ND	ND	ND	ND	ND	ND	ND	ND	0.33	30	0.61	***24***
PC021	ND	ND	ND	0.33	ND	ND	ND	ND	ND	ND	ND	ND	ND	ND	0.33	30	0.92	***30***
PC052	ND	ND	0.00	0.33	ND	ND	ND	ND	ND	ND	ND	ND	ND	ND	0.33	30	0.40	***30***
PC073	ND	ND	0.00	0.17	0.00	0.00	0.17	0.17	0.00	0.00	0.17	0.00	ND	ND	0.17	78	0.39	***66***

S2 Table: Evolution of broadly neutralizing antibody activities in the plasma of Protocol C Top neutralizers

Plasma samples collected a various time points post infection from Protocol C participants were tested for neutralization against a predictive 6 cross-clade pseudovirus panel (Simek 2011) and a medium tier1-3 pseudovirus panel (N=37, Fig S4A). Neutralization scores on each panel were calculated for each sample as detailed in the Methods section. The best 6-virus panel neutralization score across all tested time points (Best), the last tested time point (Last TP in months post infection), the 37-virus panel neutralization score (37v Score) and the time point tested on the 37-virus panel (37v TP) are also indicated. Neutralization scores are color-coded as follows: (blue) 0 < score < 0.5; (green) 0.5 ≤ score <1; (yellow) 1 ≤ score <1.5, 1.5 ≤ score <2 and (pink) 2 ≤ score <3. Participants are listed based on their best neutralization score across the study period.

S3 Table: Recombinant gp120 proteins used for absorption experiments.
Participants are listed based on their neutralization score on the 37-virus panel.

Donor	gp120 used for depletion
PC068	BG505,92RW020,94UG103,92TH021,92BR020,BaL,JRCSF,JRFL,YU2
PC094	92RW020,92BR020
PC039	92RW020,92BR020
PC002	92BR020,92TH021,IAVIC22,BG505,92RW020
PC063	JRFL,BaL
PC268	IAVIC22,JRFL
PC041	92BR020, 94UG
PC048	BaL,BG505,
PC037	JRFL
PC258	92RW020,IAVIC22
PC064	92TH021, BaL, BG505, JRCSF
PC023	92BR020
PC029	92BR020
PC025	IAVIC22,BaL
PC043	92BR020, 92RW020
PC022	92BR020
PC092	92BR020, 92RW020
PC079	92TH021, JRCSF
PC080	JRFL
PC174	92RW020,JRFL
PC036	92BR020, 92RW020
PC181	92RW020,IAVIC22
PC082	92RW020,JRCSF,BaL,IAVIC22
PC035	JRFL
PC030	IAVIC22
PC076	92BR020, 92RW020, JRCSF
PC144	BaL,92RW020
PC053	JRFL
PC178	
PC008	92RW020
PC097	BaL
PC011	IAVIC22,IAVIC22,92RW020
PC054	92BR020, 92RW020
PC241	92RW020,IAVIC22
PC031	92TH021, JRCSF
PC050	JRCSF,BG505
PC049	
PC069	92BR020
PC214	92RW020,IAVIC22
PC074	IAVIC22
PC014	JRCSF
PC017	

S4 Table: Effect of N276-glycan removal on the broad neutralizing activity of Protocol C neutralizers.

Participants are listed based on their neutralization score on the 37-virus panel.

Donor	Fold Increase in ID50 of N276A compared to WT pseudovirus					
	92RW020	94UG103	92TH021	JRFL	JRCSF	IAVIC22
PC068	1.2	0.6		1.6	6.4	1.0
PC094	0.9	1.0		1.9	2.5	0.6
PC039	1.0	0.9	0.8	0.4	0.9	1.1
PC002	1.3	0.3		35.0	2.6	0.9
PC063	10.9	1.0		18.3	0.0	18.8
PC268	1.1	0.1		1.8	1.4	0.5
PC041	1.1	0.2	0.4	0.2	0.9	0.7
PC048	1.4	0.1		8.6	1.5	5.0
PC037	1.2	1.0	0.8	0.3	0.8	0.7
PC258	1.5	0.2		2.1	1.5	1.1
PC064	0.8	0.3	1.0	0.6	1.5	1.0
PC023	2.1	1.1		2.0	0.4	0.6
PC029	1.7	1.1	1.0	0.6	2.4	1.2
PC025	1.3	0.3		1.3	1.2	1.0
PC043	1.5	0.9	1.0	0.1	5.1	0.4
PC022	0.9	1.0	1.0	0.1	1.2	0.9
PC092	1.8	0.9	0.2	0.5	1.3	0.9
PC079	1.0	1.0	1.2	0.4	1.1	0.7
PC080	0.9	1.0	5.0	0.4	3.2	1.5
PC174	2.7	1.0		2.9	48.5	0.2
PC036	1.9	0.4	0.2	0.2	1.0	0.7
PC181	1.8	0.1		2.6	1.5	0.6
PC082	1.0	0.1		0.9	2.3	1.4
PC035	1.0	1.0	0.8	0.2	1.8	0.1
PC030	0.3	0.3		3.8	2.0	0.4
PC076	1.7	1.0	0.5	0.5	0.9	1.0
PC144	1.4	2.3		1.0	1.0	4.7
PC053	46.1	58.5	10.3	1.0	7.9	8.0
PC178	2.2	0.2		28.0	4.4	0.0
PC008	2.1	1.0	0.4	1.0	1.3	0.8
PC097	1.5	0.5	0.1	0.0	1.1	0.2
PC011	0.9	1.0		1.0	0.8	0.6
PC054	1.2	1.0	1.0	0.4	2.0	1.0
PC241	1.4	1.0		2.0	1.0	0.5
PC031	0.1	1.0	0.5	0.0	0.8	0.5
PC050	0.0	0.0		1.0	3.9	0.4
PC049	1.5	1.0		1.5	0.6	0.7
PC069	1.0	1.0	0.8	0.2	0.7	1.0
PC214	1.3	1.0		1.5	0.5	0.4
PC074	0.1	1.4		0.5	7.3	0.1
PC014	1.0	1.0	0.2	0.4	1.0	0.9
PC017	0.9	0.7		1.1	1.8	0.1
PC233	1.5	1.0		1.0	1.0	0.5
PC012	1.0	1.0	1.0	0.2	5.0	0.0
PC001	2.1	2.0	0.6	0.6	2.2	0.8
PC081	1.0	1.0	1.0	1.0	2.0	1.0
PC192	1.4	1.0		1.0	1.0	1.0
PC104	0.2	0.6		1.1	0.5	0.0
PC021	1.8	1.0		2.8	1.0	0.9
PC033	1.0	1.0	0.6	0.0	1.0	2.5
PC045	1.0	1.0		1.0	1.0	0.2
PC044	0.1	1.0	0.4	0.0	2.0	0.8
PC060	0.9	1.0	1.0	0.2	1.2	0.6
PC077	0.1	0.1		1.0	2.5	0.0
PC042	1.0	0.1	1.0	0.1	5.3	0.3
PC032						
PC016	1.0	1.0	1.0	0.1	1.0	0.8
PC075	1.0	1.0	0.6	0.2	2.0	5.5
PC024	1.0	0.7	0.5	0.1	5.0	1.0
PC067	1.3	1.0	1.0	1.4	1.0	0.7
PC015	1.3	1.0	0.2	0.1	2.0	0.1
PC019	0.6	0.4	1.0	0.0	0.4	1.7
PC061	1.0	1.0		1.0	1.0	0.3
PC003	0.1	1.0		1.0	2.4	0.7
PC052						
PC051						
PC073	1.0	1.0		1.0	1.0	1.0
PC026						
